# Assessing radiation dosimetry for microorganisms in naturally radioactive mineral springs using GATE and Geant4-DNA Monte Carlo simulations

**DOI:** 10.1371/journal.pone.0292608

**Published:** 2023-10-12

**Authors:** Sofia Kolovi, Giovanna-Rosa Fois, Sarra Lanouar, Patrick Chardon, Didier Miallier, Lory-Anne Baker, Céline Bailly, Aude Beauger, David G. Biron, Karine David, Gilles Montavon, Thierry Pilleyre, Benoît Schoefs, Vincent Breton, Lydia Maigne, with the TIRAMISU Collaboration

**Affiliations:** 1 Laboratoire de Physique de Clermont (LPC) - UMR6533, CNRS/IN2P3 Université Clermont Auvergne, Aubière, France; 2 LTSER “Zone Atelier Territoires Uranifères”, Clermont-Ferrand, France; 3 Laboratoire Microorganismes: Génome Environnement (LMGE) - UMR6023, CNRS, Université Clermont Auvergne, Clermont–Ferrand, France; 4 Laboratoire de Géographie Physique et Environnementale (GEOLAB) - UMR6042, CNRS, Université Clermont Auvergne, Clermont-Ferrand, France; 5 Laboratoire de Physique Subatomique et des Technologies Associées (SUBATECH) - UMR6457, CNRS/IN2P3/IMT Atlantique/Université de Nantes, Nantes, France; 6 Metabolism, Molecular Engineering of Microalgae and Applications, Laboratoire de Biologie des Organismes, Stress, Santé Environnement, IUML FR3473, CNRS, Le Mans University, Le Mans, France; Universiti Teknologi Malaysia, MALAYSIA

## Abstract

Mineral springs in Massif Central, France can be characterized by higher levels of natural radioactivity in comparison to the background. The biota in these waters is constantly under radiation exposure mainly from the *α*-emitters of the natural decay chains, with ^226^Ra in sediments ranging from 21 Bq/g to 43 Bq/g and ^222^Rn activity concentrations in water up to 4600 Bq/L. This study couples for the first time micro- and nanodosimetric approaches to radioecology by combining GATE and Geant4-DNA to assess the dose rates and DNA damages to microorganisms living in these naturally radioactive ecosystems. It focuses on unicellular eukaryotic microalgae (diatoms) which display an exceptional abundance of teratological forms in the most radioactive mineral springs in Auvergne. Using spherical geometries for the microorganisms and based on *γ*-spectrometric analyses, we evaluate the impact of the external exposure to 1000 Bq/L ^222^Rn dissolved in the water and 30 Bq/g ^226^Ra in the sediments. Our results show that the external dose rates for diatoms are significant (9.7 μGy/h) and comparable to the threshold (10 μGy/h) for the protection of the ecosystems suggested by the literature. In a first attempt of simulating the radiation induced DNA damage on this species, the rate of DNA Double Strand Breaks per day is estimated to 1.11E-04. Our study confirms the significant mutational pressure from natural radioactivity to which microbial biodiversity has been exposed since Earth origin in hydrothermal springs.

## Introduction

Natural radioactivity has been present on Earth since its origin. A growing pattern of evidence suggests that its current levels may affect the mutational load and consequently the genetic composition of plants and animals [1, 2].

Radioactivity also plays an important role in the evolution of the terrestrial microbial biodiversity. At the bottom of mines or beneath the ocean floor, drillings reveal the presence of vast communities of microorganisms in the subsurface of our planet where water radiolysis, following the decay of radionuclides, leads to hydrogen (H_2_) and oxidants production [3]. This radiolysis could yield enough energy to fuel a large portion of this deep subsurface biome [4]. While ionising radiations have been considered toxic at any level of exposure, experiments at low radiation backgrounds provide a window to explore the contention that responses to radiation dosage are hormetic. Indeed, microbial life is stressed when it is deprived of background levels of radiation [5].

Yet, understanding the role of natural radioactivity in the evolution of microbial biodiversity is methodologically challenging due to its multi-parametric nature [2]. In this perplex context, the assessment of the radioecological risk to the environment due to ionizing radiation has been traditionally addressed through its biota since it is the sensitive component of the ecosystems. Important initiatives, such as ERICA (Environmental Risks from Ionising Contaminants: Assessment and management) tool, provide a number of assessment components following the ICRP (International Commission on Radiological Protection) approach including modelling the transfer of radionuclides through the environment, estimating dose rates to biota from internal and external distributions of radionuclides, and establishing the significance of the dose rates received by organisms [6]. The relevance of ERICA integrated approach has been demonstrated to assess the environmental risks from ionising radiation to macroscopic organisms, but the difficulty of measuring in vivo the dose rates received by biota in the size of a few micrometers in order to assess the potential radiation-induced damages at their DNA (nanoscale) makes essential the use of micro- and nano-dosimetry approaches [7, 8].

A common trend in experimental microdosimetry, as applied in a great variety of fields from aviation and space to nuclear installations and radiation therapy, is the validation of the microdetectors performance by Monte Carlo Simulations (MCS) [9–12]. As it has already been shown in the case of human cells, MCS are needed for micro- and nano-dosimetric assessements due to the stochastic nature of the energy deposition at the cell scale [13].

Aiming to cover the needs of microscale radioecology, we introduce, in this paper, a methodology for modelling the external radiation exposure and its impact on microorganisms living in naturally radioactive aquatic ecosystems using the open-source MCS tools GATE and Geant4-DNA. This work builds upon previous efforts to simulate the impact of the natural radiation background on bacterial systems in the context of very low radiation biological laboratory experiments [14]. We extend it here to eukaryotic microorganisms and to naturally radioactive aquatic ecosystems. An example of such ecosystems is mineral springs, geological formations where microorganisms have been growing in the presence of the radioisotopes of the three natural decays series (^238^U, ^232^Th, ^235^U), as well as ^40^K, since life appeared on Earth [15–18].

The natural decay series of these primordial radionuclides consist of *α*- and *β*-emitters, with the former ranging in energy between 4 and 9 MeV. ^226^Ra (t_1/2_ = 1600 y) and its gaseous descendant ^222^Rn (t_1/2_ = 3.82 d), both *α*-emitters of maximum energy 4.8 MeV and 5.5 MeV respectively [19], are found in high concentrations in the sediments (up to 31 Bq/g ^226^Ra) and waters (up to 4600 Bq/L ^222^Rn) of the mineral springs in Auvergne [35]. This volcanic region of Massif Central in France is characterized by high uranium content [20] that results in the elevated radium and radon activities measured in local waters [21, 22].

Among other microorganisms living in these peculiar ecosystems, diatoms have received particular attention in recent years. These eukaryotic, photosynthetic, unicellular microalgae are present in marine and freshwater habitats including mineral springs [23, 24] and account for a great part of the carbon dioxide fixation [25, 26]. They vary vastly in shapes and size which can range from a few μm to 2 mm in some cases [27]. What makes them unique to be encountered in the living matter is their frustule, a rigid siliceous cell wall that acts as an external skeleton, remains as fossil after their death [28, 29] allowing studies of their evolution and opens opportunities for their bionanotechnological applications [30–33]. Due to their sensitivity to environmental stresses [34], diatoms are, also, well established as water quality bio-indicators. An exceptional abundance of deformations in the most radioactive springs in Auvergne has been recently revealed, initiating studies of the effects of natural radioactivity on benthic diatom communities in 16 mineral springs of the area [35].

The goal of this paper is to use micro- and nanodosimetric MCS tools to evaluate the dose rates received by the diatoms and the potential induced DNA damage for measured activity concentrations of ^222^Rn in the water and ^226^Ra in the sediments. GATE is used for the modelling of the radioactive environment and the dose rate assessments, while Geant4-DNA is used for the prediction of Single (SSB) and Double (DSB) DNA Strand Breaks.

GATE (Geant4 Application for Tomographic Emission) is an open-source software based on Geant4 libraries, initially dedicated to medical physics, from imaging to radiotherapy and radiation protection [36–40]. Geant4 (GEometry ANd Tracking) is a simulation toolkit for the passage of particles through matter [41]. Covering a wide range of applications from radiation protection and medical physics to high energy physics, astrophysics and space science, it offers the ability of modelling and simulating from nanoscale up to macroscale [42–46]. Geant4-DNA is dedicated to the simulation of the biological damage in the DNA scale. The set of physics processes used here are adapted to micro- and nano-dosimetry in liquid water allowing the tracking of particles down to eV energies [47–50]. Among others, the assessment of the Single and Double DNA Strand Breaks due to the direct and indirect energy deposition of the ionizing particles is provided through clustering algorithms [51]. In this work, we are engaging the DBSCAN (Density Based Spatial Clustering of Applications with Noise) algorithm [52] to offer a first evaluation of the potential SSBs and DSBs on diatoms due to their chronic exposure to ionizing radiation. For the calculation of DNA damage, DBSCAN takes into account the distribution of deposited energy induced by ionising radiation (*α*-particles in our case) in micrometric geometries and a damage probability function which depends on the total deposited energy.

We first present the measured activity concentrations of the radionuclides of interest in the mineral springs and we gradually build the environmental composition which is essential for the simulation. After detailing the modelling in GATE, we focus on the dose rates received by the microorganisms considering normal benthic and extreme environmental conditions and we study the corresponding effect of the frustule. Then, we couple the GATE results with the Geant4-DNA code to perform the DNA damage simulation and we conclude with the evaluation of the predicted Single (SSB) and Double (DSB) DNA Strand Breaks.

## Materials and methods

**Fig 1:**
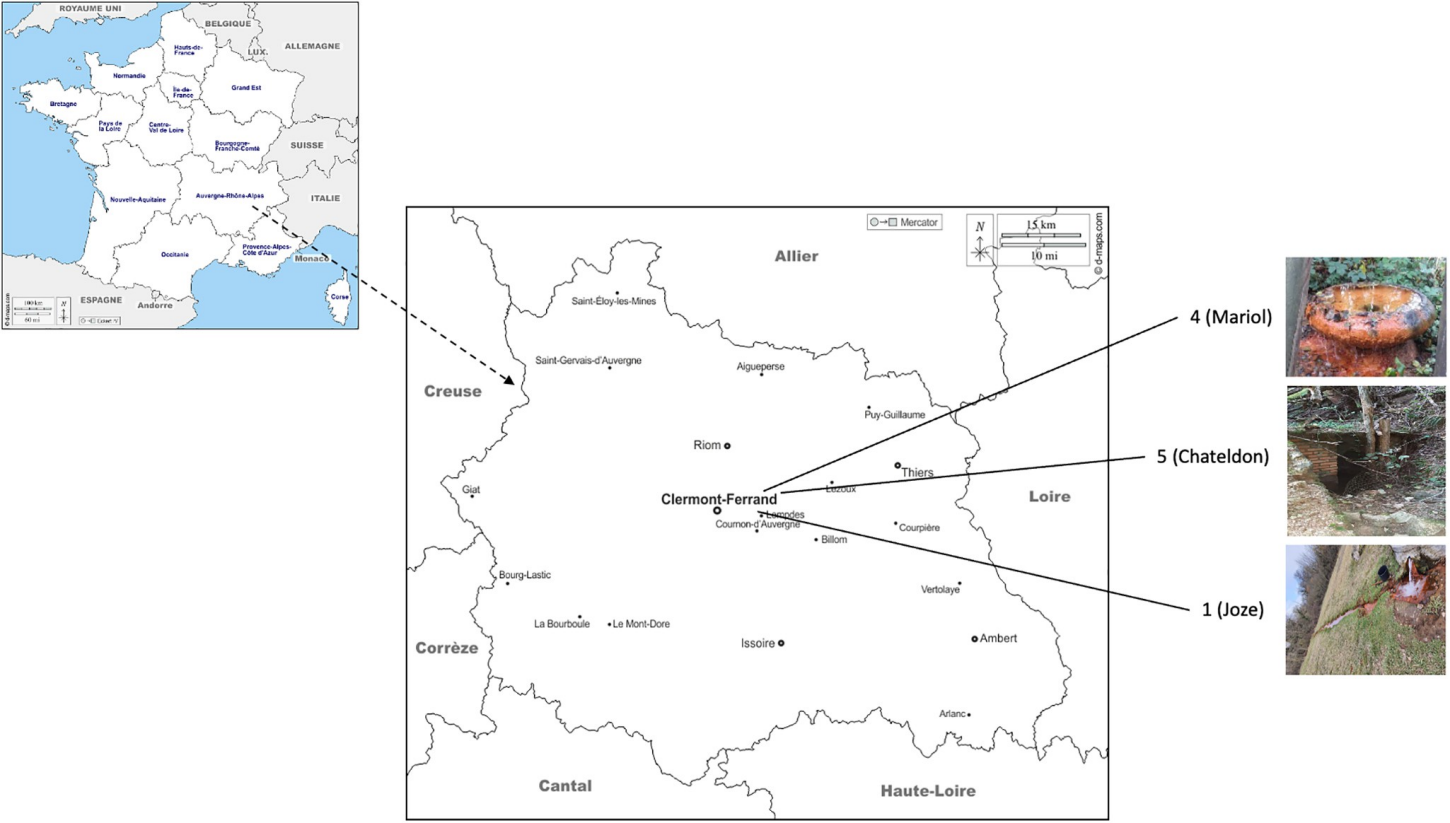
Location of springs 1 (Joze), 4 (Mariol) and 5 (Chateldon). Maps are reprinted from [53] under a CC BY license, with permission from Daniel Dalet, original copyright 2023.

The simulation study builds upon the experimental characterization of radionuclides present in water and sediments of five mineral springs in Auvergne, Massif Central, France (Tables 1 and 2). The springs are open for public use and no permit was required to access them and collect samples (Fig 1).

In two of these springs—Mariol and Chateldon—the deformation rate of different diatom species was documented as a function of the water ^222^Rn content [35]. In the following subsections, we first describe how the aquatic environment and the diatoms have been characterized, to then, elaborate the multi-scale simulations using GATE and Geant4-DNA softwares.

### Radiological characterization of the mineral springs

The activity concentrations of ^222^Rn in water and ^226^Ra in the sediments from five mineral springs were measured by *γ*-spectrometry using a High-Purity Germanium (HPGe) well-type detector (GCW3523,Canberra Inc., Toledo, USA) of 35% relative efficiency. Water was collected according to ISO 5667-1 and ISO 5667-3 standards in Marinelli style gas-analysis containers (NUVIA Instruments GMBH) designed for *γ*−spectroscopic analysis. The beakers were sealed to avoid any ^222^Rn leakage. ^222^Rn activity concentrations were measured within the first 3 hours after the sampling using the 352 keV *γ*-ray of ^214^Pb (t_1/2_ = 27.06 min). The sediment samples were dried in a laboratory fume hood until constant mass, sieved to remove parts greater than 2 mm and finally sealed, according to ISO 18589-2. By weighting the sediment sample before and after the drying process, we estimated the environmental medium to be a mixture composed by 90% water and 10% dry sediments. The measurements of the ^226^Ra mass activity were performed at least 4 weeks after the sealing to allow the secular equilibrium between ^226^Ra (t_1/2_ = 1600 y) and the immediate ^222^Rn (t_1/2_ = 3.82 d) daughters (^214^Bi—t_1/2_ = 19.9 min, ^214^Pb—t_1/2_ = 27.06 min) to be reached. The 609.3 keV *γ*-peak of ^214^Bi and the 295, 352 keV *γ*-peaks of ^214^Pb were, then, used to determine the ^226^Ra activity in the sediments.

The range of the measured activity concentrations for ^222^Rn in water and ^226^Ra in sediments are presented in Tables 1 and 2, together with the activity concentrations of ^238^U and two of the ^232^Th decay chain daughters; ^228^Ra and ^228^Th. A common feature of several CO_2_-rich geothermal systems is a high high radium content acquired during the ascent of water from the deep reservoir through the crystalline bedrock. High radium solubility in the mineral waters can be related to their major ion composition [21]. As a consequence, the sediments and deposits (travertine) precipitated from the waters display a disequilibrium in the uranium and thorium decay chains. Millan et al. [35] established a first correlation between ^222^Rn activity measured in water of 17 mineral springs in Auvergne (including the ones considered in this study) and teratological forms of diatoms. They remark that significant rates of deformations are observed for activity concentrations higher than 1000 Bq/L for ^222^Rn in water and 30 Bq/g for ^226^Ra in sediments; therefore, those two values were kept as reference for the simulation studies.

**Table 1:** Measured activity concentrations of ^222^Rn in water in five mineral springs in Auvergne (Massif central).

Spring	Coordinates	Sampling date	^222^Rn (Bq/L)
1 (Joze)	45.85057°N 3.31718°E	03/05/2017	13.7 ± 0.2
2 (Joze)	45.84927°N 3.31363°E	03/05/2017	25.3 ± 0.2
3 (Joze)	45.85008°N 3.31826°E	03/05/2017	421.6 ± 0.6
4 (Mariol)	46.02094°N 3.50589°E	13/03/2017	147.5 ± 0.9
5 (Chateldon)	45.98366°N 3.53079°E	02/03/2017	4594.0 ± 2.4

**Table 2:** Measured mass activities of radionuclides present in sediments in five mineral springs in Auvergne (Massif central).

Spring	Sampling Date	^226^Ra (Bq/g)	^238^U (Bq/g)	^228^Ra (Bq/g)	^228^Th (Bq/g)
1 (Joze)	03/05/2017	30.8 ± 0.6	3.9 ± 0.1	9.6 ± 0.4	5.5 ± 0.3
2 (Joze)	03/05/2017	42.5 ± 0.9	4.5 ± 0.1	13.9 ± 0.6	6.7 ± 0.4
3 (Joze)	03/05/2017	21.4 ± 0.4	2.3 ± 0.1	1.6 ± 0.1	1.1 ± 0.1
4 (Mariol)	23/03/2017	31.9 ± 0.6	3.7 ± 0.1	14.7 ± 0.6	3.4 ± 0.1
5 (Chateldon)	01/03/2017	31.4 ± 0.6	5.4 ± 0.2	1.5 ± 0.1	0.4 ± 0.1

An X-Ray fluorescence analysis using a Niton XL5 analyzer (Thermo Fisher Scientific Inc., Waltham, USA) was performed for the determination of the mass fraction of the mean elementary composition of the sampled sediments (Table 3). Carbon, oxygen, calcium and silicon account for approximately 95% of the mass fraction with the rest consisting of heavier metals such as iron, aluminum, potassium, magnesium, strontium and titanium, and a smaller contribution of chlorine, sulfur and phosphorus.

**Table 3:** Dry sediments composition using X-Ray fluorescence analysis.

Element	%	Element	%
C & O	60.36	K	0.70
Ca	24.50	Mg	0.53
Si	10.00	Sr	0.50
Fe	1.60	Ti	0.30
Cl	1.40	S	0.30
Al	1.00	P	0.08

### Characterization of the diatoms communities

Diatoms belong to a rich ecosystem of microorganisms including bacteria and viruses that thrive in mineral springs. The benthic species inhabit rocks or sediments of the springs floor but they can also be found in the water column. Small samples of epipelic and epilithic raw material were prepared for light microscopy (LM) observations, morphometric measurements and evaluation of the relative abundance of diatom species. Diatoms were imaged using an ultra high-resolution analytical field emission scanning electron microscope (SEM) Hitachi SU-70 (Hitachi High-Technologies Corporation, Japan). SEM images revealed various abnormal forms of diatoms and information concerning their dimensions were used to build a model for the simulation purpose. Ellipsoidal dimensions of individuals vary between 5 to 50 μm for the major axis and 4 to 7 μm for the minor axis. The study by Beauger et al. [23] provides details about 18 dominant diatom species with relative abundance higher than 1% in 17 springs located near Clermont-Ferrand close to Allier river. *Planothidium frequentissimum*, *Navicula sanctamargaritae* and *Crenotia thermalis* are three of the species with an abundance exceeding 60%. The same species are observed at spring 5 (Chateldon) where deformation rates above 25%, denoted by abnormally shaped frustules, have been observed on *Planothidium frequentissimum* [35]. Diatom deformations, or else stated teratological formations, are abnormalities on their morphology, mainly on the valve shape and structural characteristics which result in deformed frustules.

Genomics information for diatoms living in Auvergne mineral springs is currently missing. Indeed, only ten out of 200 000 diatom species have been completely sequenced up to day, revealing a range in DNA size between 27 and 162 Mbp preserved in 1—2 μm diameter nuclei [54, 55].

### Dosimetry simulation using GATE

Absorbed dose rates to a diatom have been computed with GATE v9.1 using Geant4 v.11.0.0 libraries. Being the most abundant constituent of cells, water is considered as a surrogate to the biological medium [56, 57]. In our simulation, the microorganism and its nucleus were modelled as water spheres of 10 *μ*m (r_M_) and 0.5 *μ*m radius respectively. For the modelling of the diatom, a SiO_2_ (Silicate) shell of 2 *μ*m thickness (F) was added around the microorganism, representing their frustule (rigid exoskeleton).

The environment surrounding the microorganism was modelled as a sphere with a radius (R_env_) calculated according to the following formula:

$$\begin{array}{c} R_{env}=\left(R_{max}\;+r_{M}\;+F\;\right)\cdot 1.03 \end{array}$$ (1)

where R_max_ represents the range in water of the most energetic *α*-particles in the simulation, (R_max_ = 43.44 *μ*m for ^222^Rn [58]) and the multiplication factor 1.03 offers an extra 3% space margin. The composition of the environment can be either water, dry sediments (Table 3) or a mixture of water and dry sediments, denoted as “benthic mixture”(BM). A percentage porosity (P), defined in Eq 2, is used to characterize each simulated environment:

$$\begin{array}{c} P\left(\%\right)=\frac{V_{W}}{V_{tot}}\cdot 100 \end{array}$$ (2)

where V_W_ is the volume of water and V_tot_ is the total volume of the mixture. In this work, we focus on three porosity values to define the environment: “0%”, “90%” and “100%”. The “0%” corresponds to an environment made only with dry sediments, the “100%” with only water, while “90%” porosity refers to the observed conditions at the bottom of the water column where benthic diatoms develop. Microorganisms living in the water column are typically exposed to doses corresponding to 100% porosity while the benthic ones, living on the floor of the springs or on rocks, are typically exposed to the 90% porosity scenario. It should be clear that the dry sediment scenario corresponding to 0% porosity does not reflect a relevant environment for diatoms inhabiting mineral springs and should be considered as an upper limit in this context. However, diatoms have been observed living outside water [59] and some springs dry up during summer seasons.

This study focuses on the dose rates to microorganisms coming from radioelements that have been measured experimentally to have the highest activity concentrations in the sediments and waters of Auvergne mineral springs. As a result, we simulated only the *α*-particles emitted directly by ^222^Rn and ^226^Ra (see Table 4). These radioelements also decay and their daughters, especially the *α*-emitters, contribute an additional radiation dose to the microorganisms. In a first step, this contribution was not computed using Monte-Carlo simulations because the chemical behaviour of these radionuclides and therefore their location in the vicinity of the diatoms is not known. We verified by preliminary simulations that *β*-emitters in the ^238^U decay chain, mainly ^214^Bi and ^210^Pb, could be neglected. Their contribution to the dose is 0.02% in comparison to the *α*-emitters and they were, consequently, not taken into consideration in the simulation. For each radionuclide, the *α*-particles were emitted isotropically (4*π* solid angle) from the spherical volume surrounding the microorganism where their emission point was randomly distributed. Separate simulations were performed for ^222^Rn and ^226^Ra. For pure dry sediments (0% porosity), ^226^Ra is the only source of radioactivity, while for pure water (100% porosity) only ^222^Rn is considered. Both of the radionuclides are present in the “benthic mixture” (90% porosity).

**Table 4:** Simulated *α*-particle energies and intensities from ^222^Rn [60] and ^226^Ra [61].

Radionuclide	E_*α*_ (MeV)	Intensity (%)
^222^Rn	4.826	5.0E-04
	4.986	0.078
	5.490	99.92
^226^Ra	4.160	2.7E-04
	4.191	1.0E-03
	4.340	6.5E-03
	4.601	6.16
	4.784	93.84

In all simulations we used the Geant4 electromagnetic physics list option 4. The production cuts applied to secondary electrons and gammas, which are produced due to the interactions of the *α*-particles with the matter, were investigated in preliminary simulations, and chosen to be 2 orders of magnitude less than the size of the radius of the simulated volumes: 0.1 *μ*m in the environment, 0.01 *μ*m in the diatom (microorganism plus frustule), and 0.001 *μ*m in the nucleus, corresponding thus to the lowest cut-off energy available in GATE (250 eV). The setup of the simulation is summarized in Table 5. G4_WATER was used as material in all cases [62].

**Table 5:** Summary of GATE simulation characteristics.

	environment (frustule*)	microorganism	nucleus	* frustule
**Shape**	Sphere	Sphere	Sphere	Shell
**Size**	R = 55 *μ*m (57.1 *μ*m)	R = 10 *μ*m	R = 0.5 *μ*m	Width = 2 *μ*m
**Material**	G4_WATER / dry sediments / mixture	G4_WATER	G4_WATER	Silicate
**Density (g/cm^3^)**	1.00 / 1.20 / 1.02	1.00	1.00	2.40
**Cuts (*μ*m)**	0.1	0.01	0.001	0.01
**Source**	^222^Rn / ^226^Ra	-	-	-
**Physics List**	electromagnetic standard option 4			

First, information concerning particles (energy, position, direction and particle type) entering the microorganism were recorded in a phase space (PhSp) file attached to the external boundary of the microorganism. Then, the energy deposited to the microorganism for each radionuclide was recorded, the contributions of the dominant physical processes were identified, and the total energy depositions were calculated. Absorbed dose rates to the microorganism in μGy/h were calculated for every porosity level while the silicate frustule was taken into account for the simulation of the “benthic mixture” (90% porosity). We ran separate simulations with 1E+08 primary *α*-particles for each radionuclide and repeated them 10 times to evaluate the statistical fluctuations (kept below 1%). We, then, scaled the dose rates obtained to the primaries corresponding to the reference activity values. Information concerning *α*-particles entering the nucleus were recorded in a PhSp file attached to the external boundary of the nucleus in order to be used as source description for Geant4-DNA simulations.

### Simulation of DNA damage using Geant4-DNA

The “G4EmDNAPhysics_option4” in Geant4 version 11.0.0 was used to simulate track structure of particles inside the microorganism nucleus from the PhSp file produced at the previous step. This physics list is considered the most accurate for the simulation of low energy electrons transport in water [63]. The production cuts for electrons and photons were set to 1 nm. A summary of the Geant4-DNA simulation setup is presented in Table 6.

**Table 6:** Summary of Geant4-DNA simulation characteristics.

	nucleus	nucleosomes
Dimensions	radius: 500 nm	diameter: 10 nm height: 5 nm
Material	G4_WATER	
Density (g/cm^3^)	1.00	
Cuts (nm)	1.00	
Source	*α*-particles from PhaseSpace	
Physics Lists	G4EmDNAPhysics_option4	

Using the *α*-particles generated from the PhSp file as source, we collected the distribution of deposited energies using the simplified model suggested by D.E. Charlton [64]. We simulated 30 000 water cylinders representing nucleosomes (approximately 147 base pairs each) of 10 nm diameter and 5 nm height made of water. All G4-DNA models are validated and available for water (G4_WATER as defined in NIST [62]) which is used as a surrogate to the biological medium. The cylinders were generated in random positions in the spherical nucleus of the microorganism. Energy deposits were collected in the 30 000 randomly distributed cylindrical targets and the probability distributions of the specific energies (dP/dz) over all the nucleosomes were calculated.

Specific energies usually result from several energy transfers in a given nanometric target and therefore from several physical processes. We can, however, assign a physical process to each specific energy by considering the dominant process which leads to the largest contribution of energy deposition. This allows us to study the total specific energy spectra and the different contributions from dominant processes.

The specific energy (SE) rates (μGy/h) per nucleosome were obtained by averaging the specific energies over the total number of the cylindrical targets.

To assess radiation induced SSBs and DSBs, the DBSCAN clustering algorithm was utilized [52]. It is based on the assumption that the nucleus is occupied by uniformly distributed DNA molecules, which is actually indicative of diatoms presenting an active metabolic activity [65], thus making it possible to predict the potential DNA damage without the use of sophisticated DNA geometry. Such geometry is not available for the diatoms inhabiting the mineral springs because their genome has not been sequenced yet. Because diatoms living in mineral springs are smaller than the marine diatoms sequenced, we made the assumption of a 1 μm diameter nucleus enclosing 27 Mbp of genetic material.

The formation of SSBs in this algorithm is a function of the energy deposited following a probability distribution function. For deposited energies (edep) below 5 eV the damage probability is considered zero while it increases linearly up to 37.5 eV. For edep ≥ 37.5 eV the algorithm considers that all the events can cause SSBs. The minimum number of SSBs to form a DSB is set to 2 within a radius of 3.3 nm, representing roughly the distance between 10 DNA base pairs. The indirect DNA damage due to the radicals formation after water radiolysis is taken into consideration in the free parameter “SPointProb”. It describes the probability that an interaction point is located in a sensitive area, composed by the DNA helix and a virtual aura, where both direct and indirect DNA damage can occur. Strong correlations between the genome size and phenotypic characteristics, such as nuclear and cell volume, are abundantly documented in the literature for eukaryotes [66]. As a consequence, we tested different values of the free parameter “SPointProb” in order to validate the algorithm against simulation and experimental data from the literature on human cells. For the source description, we considered the PhaseSpace and the physics list described in the previous section. We ran the simulation multiple times, achieving a relative uncertainty below 0.1%.

## Results

### Absorbed dose to microorganisms

By recovering the number of particles entering the different volumes, we observed that only 2% of the primaries emitted in the 55 μm radius environment reached the microorganism, while the presence of the frustule resulted in an extra 20% decrease in the number of particles entering the diatom. In Table 7 we summarize the recorded kinetic energies of the *α*-particles (primaries) reaching the microorganism for the different simulated environments. The most energetic primaries have a mean energy of 3.3 MeV coming from ^222^Rn dropping by 12% when considering the frustule. A similar trend is observed for ^226^Ra with a mean energy of 2.5 MeV.

**Table 7:** Kinetic energy of *α*-particles reaching the microorganism for different environments (when considering frustule, values are provided in the parentheses).

Porosity (%)	Environment	Energy (MeV)	
		mean	maximum
0	Dry sediments—^226^Ra	2.8	4.8
90	Benthic Mixture—^226^Ra (frustule)	2.8 (2.5)	4.8 (4.5)
90	Benthic Mixture—^222^Rn (frustule)	3.3 (2.9)	5.5 (5.2)
100	Water column—^222^Rn	3.3	5.5

**Fig 2:**
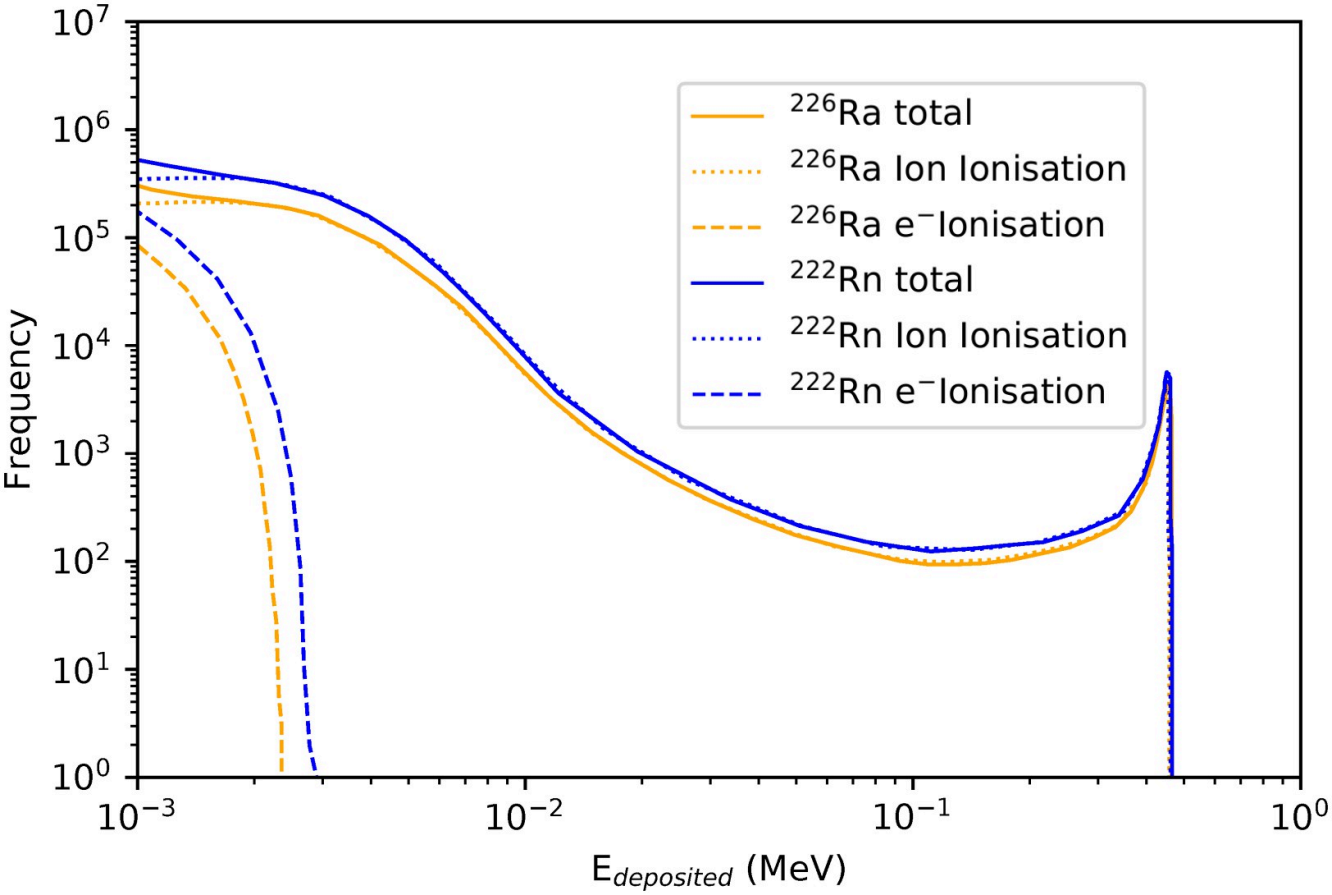
Deposited energy distributions for ^226^Ra (orange) and ^222^Rn (blue) in the benthic mixture for diatoms. Solid lines: total deposited energy, Dotted lines: ion ionisation, Dashed lines: electron ionisation.

Fig 2 shows the distribution of deposited energies to the diatom in the benthic mixture (90% porosity). The main physical processes involved in the energy depositions are ion and electron ionisations. Ion ionisation refers to the ionisations caused directly from the *α*-particle while the electron ionisation refers to the ionisations caused by sufficiently energetic secondary electrons (*δ*-rays). We show that the predominant process is ion ionisation while the mean energy deposition due to electrons ionisation is merely 15 keV.

In Table 8, the absolute doses for 1E+08 primaries are slightly higher for ^222^Rn in comparison to ^226^Ra. We remark that the frustule is responsible for an average 20% absolute dose decrease. The dose rates to the microorganisms, normalised to the reference realistic radiological conditions, are summarized in the same table. The simulation showed that a microorganism surrounded only by dry sediments (containing only ^226^Ra) would be exposed to 92.4 μGy/h whereas in the scenario of a sole aquatic environment containing only ^222^Rn the respective value gets reduced to 2.8 μGy/h.

**Table 8:** Absolute doses and normalised dose rates to microorganisms in all environments (when considering frustule, values are provided in the parentheses).

Porosity (%)	Environment	Absolute dose (Gy)	Dose Rate (μGy/h)
0	Dry sediments—^226^Ra	10.3E+04	92.4
90	Benthic Mixture—^226^Ra (frustule)	9.3E+04 (7.3E+04)	8.3 (7.4)
90	Benthic Mixture—^222^Rn (frustule)	11.1E+04 (9.2E+04)	2.5 (2.3)
100	Water column—^222^Rn	11.2E+04	2.8

Absolute doses correspond to 1E+08 primaries. Dose rates are normalised to 1000 Bq/L ^222^Rn in water and 30 Bq/g ^226^Ra in the dry sediments.

**Fig 3:**
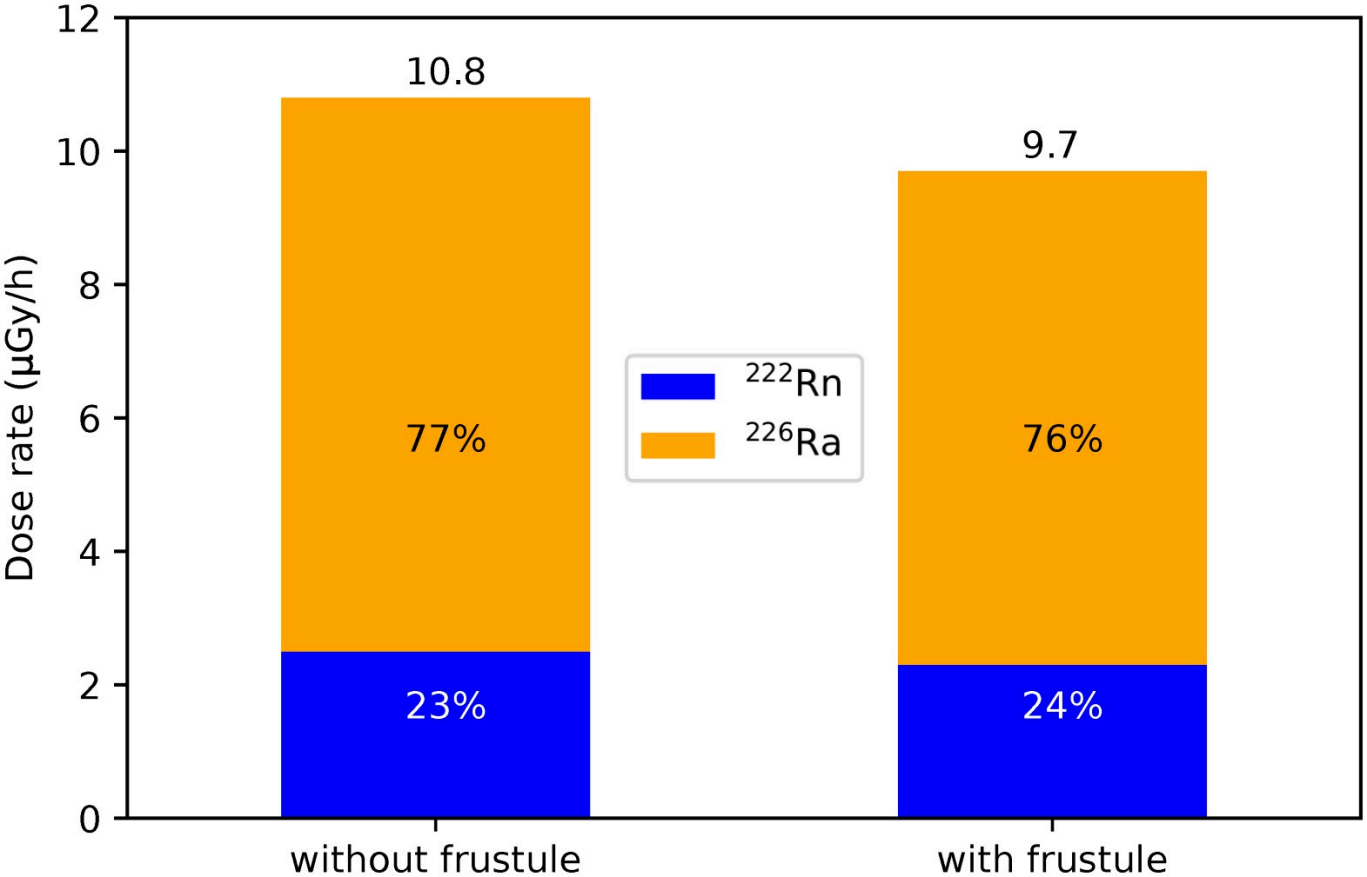
Dose rates to diatom in the benthic mixture. Blue bar: ^222^Rn contribution, Orange bar: ^226^Ra contribution.

In Fig 3 we present the dose rates to microorganisms without (10.8 μGy/h) and with frustule (9.7 μGy/h) for 1000 Bq/L ^222^Rn in water and 30 Bq/g ^226^Ra in the dry sediments. We can observe the diminution of the total dose rate due to the frustule which is equal to 10%. We highlight also that dose rates from ^222^Rn accounts only for one third of the total ones.

### DNA damage in microorganisms

From the 1E+08 primaries emitted in the environment, only very few alphas (0.003%) reached the nucleus. In Table 9 we present the mean and maximum kinetic energies of the *α*-particles entering the nucleus, as well as the mean energy they deposited to the nucleus, for the different simulated environments. The *α*-particles of ^226^Ra reach the nucleus with a 25% reduced mean energy compared to their initial emission in the environment, while with the frustule the loss is about 32%. A similar trend is observed for ^222^Rn with 18% loss. We also notice that the frustule has a very limited impact on the deposited energy.

**Table 9:** Kinetic and deposited energy of *α*-particles at the nucleus for different environments (when considering frustule, values are provided in the parentheses).

Porosity (%)	Environment	Kinetic energy (MeV)		Deposited energy (MeV)
		mean	maximum	mean
0	Dry sediments—^226^Ra	2.1	3.9	1.4E-03
90	Benthic Mixture—^226^Ra (frustule)	2.1 (1.9)	3.9 (3.5)	1.4E-03 (1.4E-03)
90	Benthic Mixture—^222^Rn (frustule)	2.7 (2.4)	4.7 (4.4)	1.3E-03 (1.3E-03)
100	Water column—^222^Rn	2.7	4.7	1.3E-03

**Fig 4:**
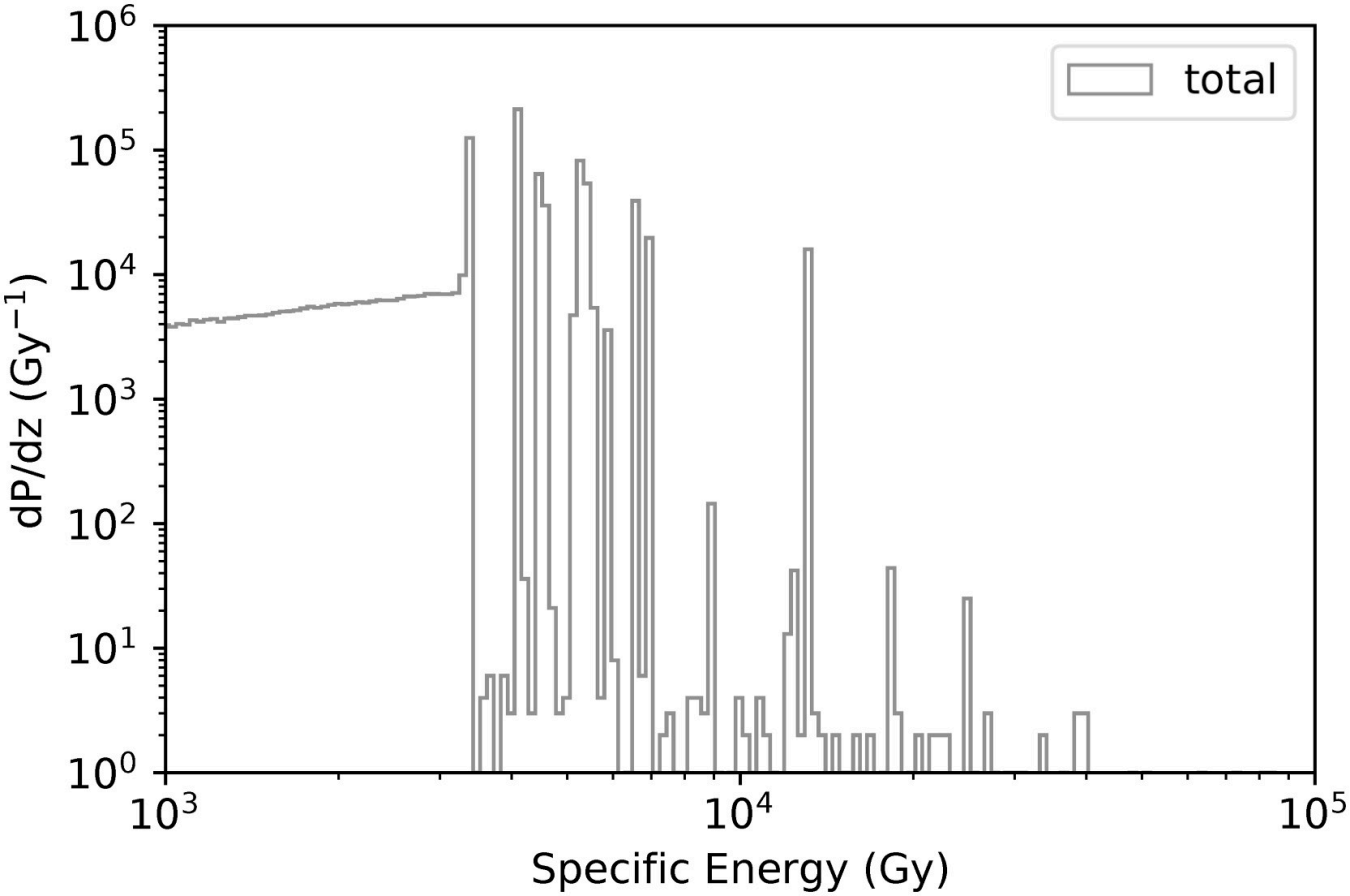
Total specific energy probability distribution (Gy^−1^) for nucleosomes (90% porosity). Grey: All processes.

**Fig 5:**
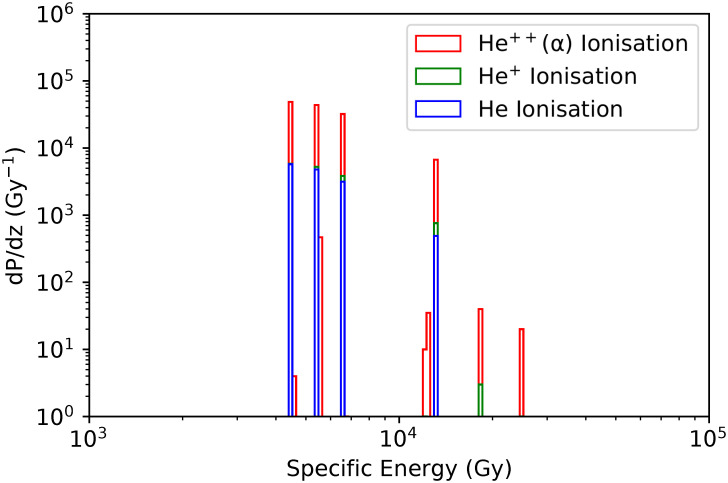
He ions specific energy probability distributions (Gy^−1^) for nucleosomes (90% porosity). Red: *α*-particles (He^+ +^) ionisation, Green: He^+^ ionisation, Blue: He ionisation.

**Fig 6:**
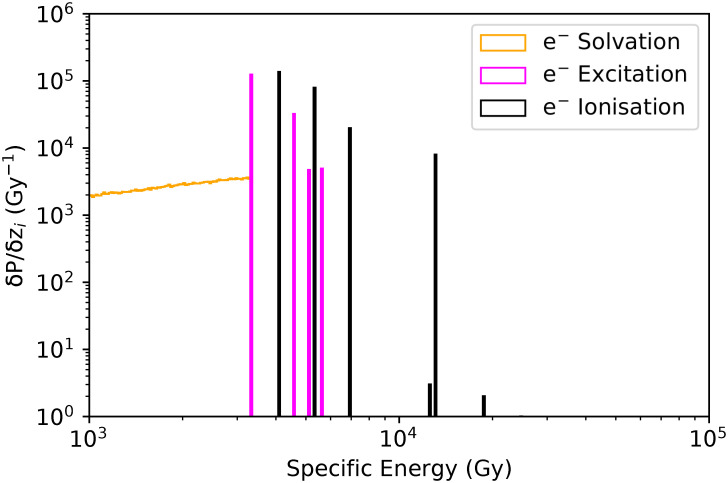
Electrons specific energy probability distributions (Gy^−1^) for nucleosomes (90% porosity). Orange: electrons solvation, Purple: electrons excitation, Black: electrons ionisation.

Fig 4 presents the total specific energy probability distribution, while Figs 5 and 6 present the distributions associated to the main physical processes in the benthic mixture (90% porosity considering the frustule). The main contributions come from He ions and electrons. We no further distinguish ^222^Rn and ^226^Ra in the benthic mixture (90% porosity) for the nanodosimetric assessment.

**Fig 7:**
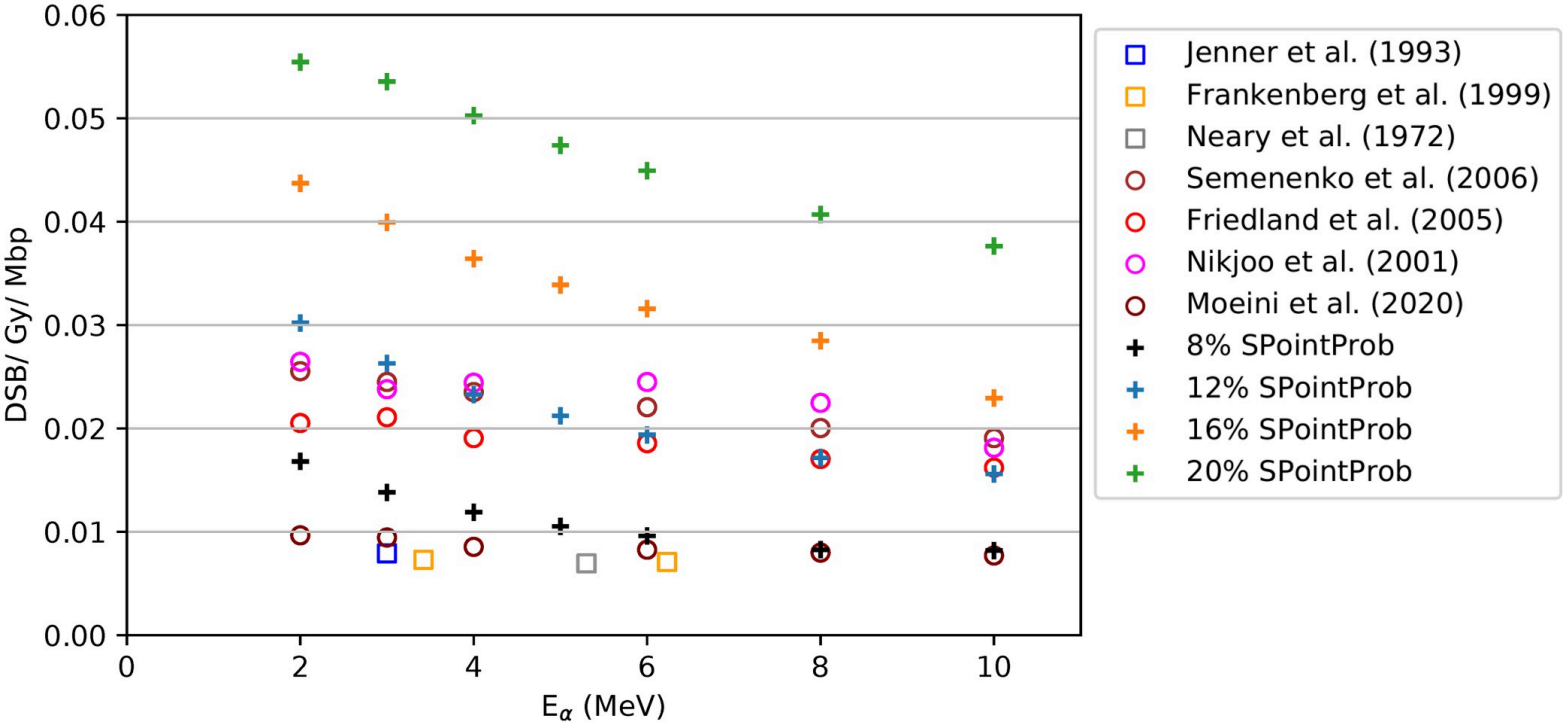
Number of DSB per Gray per Mbp as a function of the energy of *α*-particles. (+): different values of the SPointProb parameter in our work, (○): simulations using other codes found in the literature [67–70], (□): experiments found in the literature [71–73].

The DSB/Gy/Mbp for four different SPointProb values of the free parameter in the DBSCAN algorithm for monoergetic *α*-particles are plotted against the available literature [67] in Fig 7. As it is shown, our predicted values are in general agreement with the experimental and other simulation results using different MC codes. The overprediction of DSBs when using the suggested values of 16% [52] and 20% [48] smooths when lowering the value. 12% offers a good agreement with H. Moeini et al. [67] but we finally chose SpointProb equal to 8% due to the better agreement with the experimental results, too.

In Table 10, the mean specific energy (SE) rates to a nucleosome and the SSBs and DSBs obtained per Gray and per Mbp using the DBSCAN clustering algorithm (8% SPointProb) for the different simulated environments are listed. In the same table, we present the SSBs and DSBs per day normalised to 27 Mbp and, to 1 day-exposure to 30 Bq/g ^226^Ra in dry sediments and 1000 Bq/L ^222^Rn in water.

**Table 10:** Mean specific energy (SE) rates per nucleosome (μGy/h) and number of SSBs, DSBs (when considering frustule, values are provided in the parentheses).

Porosity (%)	0	90	100
Environment	Dry sediments—^226^Ra	Benthic Mixture—^226^Ra & ^222^Rn (frustule)	Water column—^222^Rn
**SE rate (μGy/h)**	71.70	8.31 (7.36)	2.12
**SSB/Gy/Mbp**	0.07	0.16 (0.15)	0.08
**DSB/Gy/Mbp**	0.02	0.03 (0.03)	0.02
**SSB/day**	4.50E-03	5.40E-04 (4.70E-04)	1.48E-04
**DSB/day**	1.06E-03	1.21-04 (1.11E-04)	2.99E-05

The SSB/day and DSB/day are normalised for 1-day exposure to 30 Bq/g ^226^Ra, 1000 Bq/L ^222^Rn and 27 Mbp DNA length

We observe that the frustule decreases the mean specific energy rates by 11%. We also highlight that the nucleosomes of microorganisms living in dry sediments containing exclusively 30 Bq/g of ^226^Ra are exposed to 34 times higher specific energies rates than the microorganisms living in water containing only 1000 Bq/L of ^222^Rn.

We, also, observe that the highest number of SSBs/Gy/Mbp and DSBs/Gy/Mbp is predicted for the benthic mixture, while the frustule decreases the values by 6%. The number of SSBs/Gy/Mbp originating solely from ^222^Rn in the water is 14% higher than from ^226^Ra in the dry sediments. When we normalize the SSBs and DSBs for 27 Mbp and 1-day exposure to 30 Bq/g of ^226^Ra and 1000 Bq/L of ^222^Rn, we observe the opposite trend; the number of SSBs/day originating from ^222^Rn in the water is 97% lower than from ^226^Ra in the dry sediments.

## Discussion

### Absorbed dose to microorganisms

The goal of this work was to apply for the first time micro- and nanodosimetric approaches and tools to evaluate the doses received by microorganisms living in naturally radioactive ecosystems. We simulated separately the *α*-particles of ^222^Rn in the water column and ^226^Ra in the sediments of mineral springs in Auvergne, excluding their daughter nuclei in a first stage. Indeed, the contribution to the dose rates coming from the radioelements in the decay chain of ^226^Ra and ^222^Rn, especially the *α*-emitters, depends on their location on the vicinity of the diatoms. Additional data are needed to understand their chemical behaviour and therefore their contribution to the dose rates to the microorganisms.

Diatoms were selected as our model organism because they were observed to display deformations in the most radioactive spring studied in Auvergne. Their frustule, composed of amorphous silicate, has been considered in the simulations in order to evaluate its impact on the absorbed dose rate compared to other microorganisms. We remind that for the purpose of this paper we considered only an external radiative environment, excluding the uptake of radionuclides through the pores of the frustule through which nutrients present in the water are absorbed.

As shown in Table 7, the interactions of *α*-particles with the 2 μm thickness frustule, with a density 2.4 times higher than that of the water, decrease the maximum kinetic energy of the particles entering the diatom by 6% on average. The impact of the frustule is also observed in the absorbed dose rates to diatoms which are 10% lower than for other microorganisms in the same environmental conditions.

As shown in Fig 3, the energy deposited from ^222^Rn (E_*α*,*max*_ = 5.5 MeV, maximum range in water = 43.4 μm) is slightly higher than the one from ^226^Ra (E_*α*,*max*_ = 4.8 MeV, maximum range in water = 35 μm). Therefore, *α*-particles will deposit a great part of their energy in the microorganism (10 μm radius) through ionisations, reaching the maximum energy deposition at the end of their range (peak observed at higher energies). Considering the same number of primaries generated for both radionuclides, the absolute dose calculated to the diatom is 26% higher for ^222^Rn than for ^226^Ra.

When dose rates are normalized to the reference activity concentrations in the environment (30 Bq/g of ^226^Ra in sediments and 1000 Bq/L of ^222^Rn in the water column), we reach 92.4 μGy/h when considering only dry sediments, 2.8 μGy/h considering only water, and 9.7 μGy/h considering both ^222^Rn and ^226^Ra contributions in the benthic mixture (see Table 8 and Fig 3). To our knowledge, dose rates to diatoms have so far been poorly documented; we could only compare to the work of Morthekai et al. [74], who investigated luminescence dating on diatom fossils in core sediments of a river and a lake. They obtained a range of values between 0.5 and 1 μGy/h due to U, Th and K. Although the environments and radionuclides differ from our work, Morthekai’s values are coherent with our calculations considering that 1.1 μGy/h is deposited at the frustule in the benthic mixture.

Moreover, it is interesting to compare our dose rate values to those recommended by the ERICA risk assessment tool [6] for the protection of the environment. ERICA provides a 10 μGy/h dose rate threshold for all the ecosystems and non-human species. Below this threshold, the environmental risks are considered negligible.

This limit value is almost reached for the benthic conditions considered in our study while it is crossed for the activities measured in spring 5 (Chateldon—values shown in Tables 1 and 2). Indeed, the dose rate to benthic diatoms in spring 5 coming only from external ^226^Ra and ^222^Rn is 18.3 μGy/h. Additional exposure is expected to come from other radioelements present either outside the diatom frustule or internally incorporated [75]. The disequilibrium observed in the ^238^U and ^232^Th decay chains confirms that the dose rate calculations should not be performed under the equilibrium hypothesis but rather according to the measured activity concentrations, as adopted by ERICA.

### DNA damage in microorganisms

As presented in Table 7, whatever the considered environment, the mean energy of *α*-particles reaching the nucleus is around 2.3 MeV corresponding to a range of 13 μm, 13 times higher than the nucleus diameter considered (1 μm). Consequently, a very small fraction of energy (around 0.07%) is deposited to the nucleus. When considering the frustule, the mean kinetic energy of *α*-particles is reduced by 10% maximum.

As shown in Figs 4–6, at nanoscale, the dominant processes in terms of probabilities are *α*-particles ionisations and excitations, as well as, ionisations, excitations and solvations of secondary electrons. Solvated electrons (free electrons in liquid water), known to play an important role in the damaging effects to the DNA [76, 77], are contributing to 15% of the total specific energy, while electrons and *α*-particles ionisations contribution to the total specific energy is 32.5% and 19.6% respectively.

From Table 10, we can remark that the frustule reduces the SE rates to a nucleosome by 11% which is very coherent with the reduction of the kinetic energy observed. The highest SE rates are obtained for 0% porosity (30 Bq/g of ^226^Ra in the dry sediments) with a 147 bp DNA receiving 34 times higher SE rates in comparison to an environment characterized by 100% porosity (1000 Bq/L of ^222^Rn in the water).

For the DNA damage assessment, we first performed a validation of the proper SPointProb value to be used in DBSCAN algorithm. The algorithm has already been validated for protons using 16% [52] and 20% SPointProb [48] to fit respective experimental and simulation data. For *α*-particles in the energy range of 2—10 MeV, we tested 20%, 16%, 12% and 8% SPointProb in an effort to best fit our results with Moeini et al. including the literature provided [67]. As we can see in Fig 7, our values of DSB/Gy/Mbp for the chosen 8% SPointProb lie within the simulation and experimental data found in the literature. After the validation, we were able to predict that the highest number of SSBs/Gy/Mbp and DSBs/Gy/Mbp takes place in the benthic mixture (90% porosity) where the effect of the frustule is no more evident.

Table 10 displays the number of SSBs and DSBs for 1-day exposure to 30 Bq/g of ^226^Ra and 1000 Bq/L of ^222^Rn considering a 27 Mbp genome. We highlight that the number of Single DNA strand breaks can differ by 1 order of magnitude from 4.50E-03 SSB / day for 0% porosity to 1.48E-04 SSB / day for 100% porosity. When Lampe et al. [78] conducted similar studies for the DNA damage induced on the prokaryotic *Escherichia coli* due to the natural background radiation, they observed that the natural radiation background near the surface was responsible for only 2.8E-08 DSB/day. As *E. coli* spontaneous mutation rate from endogenous causes is orders of magnitude higher (1.0E-03 / cell division) [14], they concluded that the background radiation had likely only a very small mutational effect on the biological system under study. With our work, we show that the computed mutation rate (4.7E-04 DSB/day) for the diatoms in the studied mineral springs is 4 orders of magnitude higher than for bacteria exposed solely to natural background radiation (2.8E-08 DSB/day) as calculated by Lampe et al. [78].

Recently, experiments to evaluate the spontaneous mutation rate in the model diatom *Phaeodactylum tricornutum* were conducted for the first time revealing a total spontaneous nuclear mutation rate per generation of approximately 1.29E-02 (accounting for base substitution and insertion-deletion mutation rates) [79].

When comparing these numbers to the rate of radiation induced SSBs and DSBs deduced from Table 10, respectively 4.70E-04 and 1.11E-04 per generation day for the benthic mixture, we observe that radiation induced mutations could contribute to an accountable mutational pressure. Our result can be related to the observed correlation between natural bedrock radioactivity and the mutation rate of waterlices living in subterranean habitants [2].

This result suggests that natural radioactivity can be an important abiotic driver of the evolution of microorganisms living in mineral springs. The comparison has of course some limitations. First, laboratory conditions impose their own sources of stress with a potential effect on the mutation rates. As a consequence, differences are expected between the experimental mutation rates and the long-term average mutation rates in the natural environment. The other limitation lies in the comparison between radiation induced damages and spontaneous mutation rates. Between the two observables lies the complete cell repair process.

## Conclusion

In this study, we focused on the simulation of the external radiation exposure of microorganisms and diatoms living in naturally radioactive aquatic ecosystems. Three different environmental compositions were simulated corresponding to the ecosystems of mineral springs: dry sediments (0% porosity) containing only 30 Bq/g ^226^Ra, water (100% porosity) containing only 1000 Bq/L ^222^Rn and a benthic mixture of both (90% porosity) representing realistic conditions. In the benthic mixture, the diatom is exposed to 9.7 μGy/h due to ^226^Ra and ^222^Rn, a dose rate which is comparable with the threshold (10 μGy/h) for the protection of the ecosystems suggested by ERICA risk assessment tool. We evaluated that the frustule does not considerably protect diatoms from ionizing radiation. Based on our computed DSB, we show that the microorganisms are extremely exposed to DNA damages due to the chronic exposure to ionising radiation in the radioactive mineral springs. By demonstrating the coupling of experimental measurements with Monte Carlo simulations for two radionuclides, this work can be implemented in future radioecological studies wishing to estimate not only dose rates but also potential DNA damages on aquatic microorganisms and extend to other radioisotopes.

## References

[1] Moller AP, Mousseau TA. The effects of natural variation in background radioactivity on humans, animals and other organisms. Biological Reviews. 2013;88(1):226–254. doi: 10.1111/j.1469-185X.2012.00249.x 23136873

[2] Saclier N, Chardon P, Malard F, Konecny-Dupré L, Eme D, Bellec A, et al. Bedrock radioactivity influences the rate and spectrum of mutation. eLife. 2020;9:1–20. doi: 10.7554/eLife.56830 33252037 PMC7723406

[3] Blair CC, D’Hondt S, Spivack AJ, Kingsley RH. Radiolytic hydrogen and microbial respiration in subsurface sediments. Astrobiology. 2007;7(6):951–970. doi: 10.1089/ast.2007.0150 18163872

[4] Sherwood Lollar B, Heuer VB, McDermott J, Tille S, Warr O, Moran JJ, et al. A window into the abiotic carbon cycle—Acetate and formate in fracture waters in 2.7 billion year-old host rocks of the Canadian Shield. Geochimica et Cosmochimica Acta. 2021;294:295–314. doi: 10.1016/j.gca.2020.11.026

[5] Castillo H, Schoderbek D, Dulal S, Escobar G, Wood J, Nelson R, et al. Stress induction in the bacteria Shewanella oneidensis and Deinococcus radiodurans in response to below-background ionizing radiation. International Journal of Radiation Biology. 2015;91(9):749–756. doi: 10.3109/09553002.2015.1062571 26073528

[6] Brown JE, Alfonso B, Avila R, Beresford NA, Copplestone D, Pröhl G, et al. The ERICA Tool. Journal of Environmental Radioactivity. 2008;99(9):1371–1383. doi: 10.1016/j.jenvrad.2008.01.008 18329765

[7] Rossi HH, Zaider M. Microdosimetry and Its Applications. SpringerLink; 1996.

[8] Rabus H. Nanodosimetry—on the”tracks” of biological radiation effectiveness. Zeitschrift fur Medizinische Physik. 2020;30(2):91–94. doi: 10.1016/j.zemedi.2020.01.002 32029295

[9] Waker AJ. Techniques for radiation measurements: Microdosimetry and dosimetry. Radiation Protection Dosimetry. 2006;122(1-4):369–373. doi: 10.1093/rpd/ncl497 17223638

[10] Payervand F, Raisali GR, Hajiesmaeilbaigi F, Saramad S. Determination of dose-equivalent response of a typical diamond microdosimeter in space radiation fields. Iranian Journal of Medical Physics. 2018;15(1):39–47. doi: 10.22038/ijmp.2017.24503.1242

[11] Braby LA. Experimental microdosimetry: History, applications and recent technical advances. Radiation Protection Dosimetry. 2015;166(1-4):3–9. doi: 10.1093/rpd/ncv137 25877539

[12] Guardiola C, Fleta C, Quirion D, Pellegrini G, Gómez F. Silicon 3D microdetectors for microdosimetry in hadron therapy. Micromachines. 2020;11(12):1–20. doi: 10.3390/mi11121053 33260634 PMC7760635

[13] Huang CY, Guatelli S, Oborn BM, Allen BJ. Microdosimetry for targeted alpha therapy of cancer. Computational and Mathematical Methods in Medicine. 2012;2012. doi: 10.1155/2012/153212 22988479 PMC3439982

[14] Lampe N, Biron DG, Brown JMC, Incerti S, Marin P, Maigne L, et al. Simulating the impact of the natural radiation background on bacterial systems: Implications for very low radiation biological experiments. PLoS ONE. 2016;11(11):1–19. doi: 10.1371/journal.pone.0166364 27851794 PMC5112919

[15] Pirajno F. Subaerial hot springs and near-surface hydrothermal mineral systems past and present, and possible extraterrestrial analogues. Geoscience Frontiers. 2020;11(5):1549–1569. doi: 10.1016/j.gsf.2020.04.001

[16] Kitto ME, Parekh PP, Torres MA, Schneider D. Radionuclide and chemical concentrations in mineral waters at Saratoga Springs, New York. Journal of Environmental Radioactivity. 2005;80(3):327–339. doi: 10.1016/j.jenvrad.2004.10.006 15725506

[17] Saqan SA, Kullab MK, Ismail AM. Radionuclides in hot mineral spring waters in Jordan. Journal of Environmental Radioactivity. 2001;52(1):99–107. doi: 10.1016/S0265-931X(00)00096-5 11202689

[18] Djokic T, VanKranendonk MJ, Campbel KA, Walter MR, Ward CR. Earliest signs of life on land preserved in ca. 3.5 Ga hot spring deposits. Nature Communications. 2017;8(May):1–8. doi: 10.1038/ncomms15263 PMC5436104 28486437

[19] NNDC National Nuclear Data Center—Online Data Service, *NuDat3*;. Available from: https://www.nndc.bnl.gov/nudat3/indx_dec.jsp.

[20] Ielsch G, Cuney M, Buscail F, Rossi F, Leon A, Cushing ME. Estimation and mapping of uranium content of geological units in France. Journal of Environmental Radioactivity. 2017;166:210–219. doi: 10.1016/j.jenvrad.2016.05.022 27266726

[21] Rihs S, Condomines M, Fouillac C. U- and Th-series radionuclides in CO2-rich geothermal systems in the French Massif Central. Journal of Radioanalytical and Nuclear Chemistry. 1997;226(1-2):149–157. doi: 10.1007/BF02063640

[22] Ielsch G, Thiéblemont D, Labed V, Richon P, Tymen G, Ferry C, et al. Radon (222Rn) level variations on a regional scale: Influence of the basement trace element (U, Th) geochemistry on radon exhalation rates. Journal of Environmental Radioactivity. 2001;53(1):75–90. doi: 10.1016/S0265-931X(00)00106-5 11378929

[23] Beauger A, Voldoire O, Wetzel CE, Allain E, Millan F, Breton V, et al. Biodiversity and ecology of diatoms in mineral springs of the area of of Sainte Marguerite (Saint-Maurice-ès-Allier, Massif central, France). BIOM. 2020;1:21–34. doi: 10.18145/biom.v1i1.250

[24] Lai GG, Beauger A, Wetzel CE, Padedda BM, Voldoire O, Lugliè A, et al. Diversity, ecology and distribution of benthic diatoms in thermo-mineral springs in Auvergne (France) and Sardinia (Italy). PeerJ. 2019;7. doi: 10.7717/peerj.7238 31341731 PMC6637927

[25] Field C, Behrenfeld M, Randerson J, Falkowski P. Primary production of the biosphere: integrating terrestrial and oceanic components. Science. 1998;281:237–240. doi: 10.1126/science.281.5374.237 9657713

[26] Scarsini M, Marchand J, Manoylov KM, Schoefs B. Photosynthesis in Diatoms. In: Seckbach J, Gordon R, editors. Diatoms: Fundamentals & Applications. Beverly, MA, USA: Wiley-Scronever; 2019. p. 191–211.

[27] Malviya S, Scalco E, Audic S, Vincent F, Veluchamy A, Poulain J, et al. Insights into global diatom distribution and diversity in the world’s ocean. Proceedings of the National Academy of Sciences of the United States of America. 2016;113(11):E1516–E1525. doi: 10.1073/pnas.1509523113 26929361 PMC4801293

[28] Sims PA, Mann DG, Medlin LK. Evolution of the diatoms: Insights from fossil, biological and molecular data. Phycologia. 2006;45(4):361–402. doi: 10.2216/05-22.1

[29] Harwood DM, Nikolaev V, Winter DM. Cretaceous records of diatom evolution, radiation and expansion. The Paleolontogical Society Papers. 2007;13:33–59. doi: 10.1017/S1089332600001455

[30] Parkinson J, Gordon R. Beyond micromachining: The potential of diatoms. Trends in Biotechnology. 1999;17(5):190–196. doi: 10.1016/S0167-7799(99)01321-9 10322443

[31] Kröger N, Poulsen N. Diatoms—From cell wall biogenesis to nanotechnology. Annual Review of Genetics. 2008;42:83–107. doi: 10.1146/annurev.genet.41.110306.130109 18983255

[32] Wang Y, Cai J, Jiang Y, Jiang X, Zhang D. Preparation of biosilica structures from frustules of diatoms and their applications: Current state and perspectives. Applied Microbiology and Biotechnology. 2013;97(2):453–460. doi: 10.1007/s00253-012-4568-0 23179621

[33] Khan MJ, Singh N, Mishra S, Ahirwar A, Bast F, Varjani S, et al. Impact of light on microalgal photosynthetic microbial fuel cells and removal of pollutants by nanoadsorbent biopolymers: Updates, challenges and innovations. Chemosphere. 2022;288:132589. doi: 10.1016/j.chemosphere.2021.132589 34678344

[34] Stevenson RJ, Pan Y, van Dam H. Assessing environmental conditions in rivers and streams with diatoms. The Diatoms: Applications for the Environmental and Earth Sciences, Second Edition. 2010; p. 57–85. doi: 10.1017/CBO9780511763175.005

[35] Millan F, Izere C, Breton V, Voldoire O, Biron DG, Wetzel CE, et al. The effect of natural radioactivity on diatom communities in mineral springs. Botany Letters. 2020;167(1):95–113. doi: 10.1080/23818107.2019.1691051

[36] Jan S, Santin G, Strul D, Staelens S, Assié K, Autret D, et al. GATE: a simulation toolkit for PET and SPECT. Physics in Medicine & Biology. 2004;4543(19):4543. doi: 10.1088/0031-9155/49/19/007 PMC3267383 15552416

[37] Jan S, Benoit D, Becheva E, Carlier T, Cassol F, Descourt P, et al. GATE V6: A major enhancement of the GATE simulation platform enabling modelling of CT and radiotherapy. Physics in Medicine and Biology. 2011;56(4):881–901. doi: 10.1088/0031-9155/56/4/001 21248393

[38] Bongrand A, Koumeir C, Villoing D, Guertin A, Haddad F, Métivier V, et al. A monte carlo determination of dose and range uncertainties for preclinical studies with a proton beam. Cancers. 2021;13(8). doi: 10.3390/cancers13081889 33920758 PMC8071150

[39] Wang Y, Li Z, Zhang A, Gu P, Li F, Li X, et al. Microdosimetric calculations by simulating monoenergetic electrons in voxel models of human normal individual cells. Radiation Physics and Chemistry. 2020;166(July 2019):108518. doi: 10.1016/j.radphyschem.2019.108518

[40] Papadimitroulas P. Dosimetry applications in GATE Monte Carlo toolkit. Physica Medica. 2017;41:136–140. doi: 10.1016/j.ejmp.2017.02.005 28236558

[41] Agostinelli S, Allison J, Amako K, Apostolakis J, Araujo H, Arce P, et al. GEANT4—A simulation toolkit. Nuclear Instruments and Methods in Physics Research, Section A: Accelerators, Spectrometers, Detectors and Associated Equipment. 2003;506(3):250–303. doi: 10.1016/S0168-9002(03)01368-8

[42] Allison J, Amako K, Apostolakis J, Araujo H, Dubois PA, Asai M, et al. Geant4 developments and applications. IEEE Transactions on Nuclear Science. 2006;53(1):270–278. doi: 10.1109/TNS.2006.869826

[43] Allison J, Amako K, Apostolakis J, Arce P, Asai M, Aso T, et al. Recent developments in GEANT4. Nuclear Instruments and Methods in Physics Research, Section A: Accelerators, Spectrometers, Detectors and Associated Equipment. 2016;835:186–225. doi: 10.1016/j.nima.2016.06.125

[44] Incerti S, Douglass M, Penfold S, Guatelli S, Bezak E. Review of Geant4-DNA applications for micro and nanoscale simulations. Physica Medica. 2016;32(10):1187–1200. doi: 10.1016/j.ejmp.2016.09.007 27659007

[45] Ivantchenko AV, Ivanchenko VN, Molina JMQ, Incerti SL. Geant4 hadronic physics for space radiation environment. International Journal of Radiation Biology. 2012;88(1-2):171–175. doi: 10.3109/09553002.2011.610865 21830895

[46] Freudenberg R, Wendisch M, Kotzerke J. Geant4-Simulations for cellular dosimetry in nuclear medicine. Zeitschrift fur Medizinische Physik. 2011;21(4):281–289. doi: 10.1016/j.zemedi.2011.08.003 21983023

[47] Incerti S, Kyriakou I, Bernal MA, Bordage MC, Francis Z, Guatelli S, et al. Geant4-DNA example applications for track structure simulations in liquid water: A report from the Geant4-DNA Project. Medical Physics. 2018;45(8):e722–e739. doi: 10.1002/mp.13048 29901835

[48] Bernal MA, Bordage MC, Brown JMC, Davídková M, Delage E, El Bitar Z, et al. Track structure modeling in liquid water: A review of the Geant4-DNA very low energy extension of the Geant4 Monte Carlo simulation toolkit. Physica Medica. 2015;31(8):861–874. doi: 10.1016/j.ejmp.2015.10.087 26653251

[49] Incerti S, Ivanchenko A, Karamitros M, Mantero A, Moretto P, Tran HN, et al. Comparison of GEANT4 very low energy cross section models with experimental data in water. Medical Physics. 2010;37(9):4692–4708. doi: 10.1118/1.3476457 20964188

[50] Incerti S, Baldacchino G, Bernal M, Capra R, Champion C, Francis Z, et al. The Geant4-DNA project The Geant4-DNA collaboration. International Journal of Modeling, Simulation, and Scientific Computing. 2010;1(2):157. doi: 10.1142/S1793962310000122

[51] Francis Z, Stypczynska A. Clustering algorithms in radiobiology and DNA damage quantification. Data Security, Data Mining and Data Management: Technologies and Challenges. 2013;(May 2013):1–29.

[52] Francis Z, Villagrasa C, Clairand I. Simulation of DNA damage clustering after proton irradiation using an adapted DBSCAN algorithm. Computer Methods and Programs in Biomedicine. 2011;101(3):265–270. doi: 10.1016/j.cmpb.2010.12.012 21232812

[53] d-maps. Available from: https://d-maps.com/index.php?lang=en.

[54] Bhattacharjya R, Tiwari A, Marella TK, Bansal H, Srivastava S. New paradigm in diatom omics and genetic manipulation. Bioresource Technology. 2021;325(January):124708. doi: 10.1016/j.biortech.2021.124708 33487514

[55] Heydarizadeh P, Marchand J, Chenais B, Sabzalian MR, Zahedi M, Moreau B, et al. Functional investigations in diatoms need more than a transcriptomic approach. Diatom Research. 2014;29(1):75–89. doi: 10.1080/0269249X.2014.883727

[56] Nikjoo H, Uehara S, Emfietzoglou D, Cucinotta FA. Track-structure codes in radiation research. Radiation Measurements. 2006;41(9):1052–1074. doi: 10.1016/j.radmeas.2006.02.001

[57] Dingfelder M. Track-structure simulations for charged particles. Health physics. 2012;103(5):590–595. doi: 10.1097/HP.0b013e3182621292 23032889 PMC3883502

[58] NIST National Institut of Standards and Technology, *ASTAR*;. Available from: https://physics.nist.gov/PhysRefData/Star/Text/ASTAR.html.

[59] Bertrand J, Coste C, Cohu RL, Renon Jp, Ector L. Étude préliminaire sur la présence de diatomées sur les lichens. Botany Letters. 2016;8107:1–23. doi: 10.1080/23818107.2016.1156573

[60] Singh B, Basunia MS, Martin M, McCutchan EA, Bala I, Caballero-Folch R, et al. Nuclear Data Sheets for A = 218. Nuclear Data Sheets. 2019;160:405–471. doi: 10.1016/j.nds.2019.100524

[61] Singh S, Jain AK, Tuli JK. Nuclear Data Sheets for A = 222. Nuclear Data Sheets. 2011;112(11):2851–2886. doi: 10.1016/j.nds.2011.10.002

[62] NIST National Institut of Standards and Technology *Materials Database*. Available from: https://materialsdata.nist.gov/.

[63] Kyriakou I, Incerti S, Francis Z. Technical note: Improvements in geant 4 energy-loss model and the effect on low-energy electron transport in liquid water. Medical Physics. 2015;42(7):3870–3876. doi: 10.1118/1.4921613 26133588

[64] Charlton DE. The deposition of energy in small cylindrical targets by high LET radiations. Radiation Protection Dosimetry. 1985;13(1-4):123–125. doi: 10.1093/rpd/13.1-4.123

[65] Stȩpiński D. Functional ultrastructure of the plant nucleolus. Protoplasma. 2014;251(6):1285–1306. doi: 10.1007/s00709-014-0648-6 24756369 PMC4209244

[66] Cavalier-Smith T. Sceletal DNA and the evolution of genome size. Ann Rev Biophy Bioeng. 1982;(11):273–302. doi: 10.1146/annurev.bb.11.060182.001421 7049065

[67] Moeini H, Mokari M, Alamatsaz MH, Taleei R. Calculation of the initial DNA damage induced by alpha particles in comparison with protons and electrons using Geant4-DNA. International Journal of Radiation Biology. 2020;96(6):767–778. doi: 10.1080/09553002.2020.1730015 32052675

[68] Semenenko VA, Stewart RD. Fast Monte Carlo simulation of DNA damage formed by electrons and light ions. Physics in Medicine and Biology. 2006;51(7):1693–1706. doi: 10.1088/0031-9155/51/7/004 16552098

[69] Friedland W, Dingfelder M, Jacob P, Paretzke HG. Calculated DNA double-strand break and fragmentation yields after irradiation with He ions. Radiation Physics and Chemistry. 2005;72(2-3):279–286. doi: 10.1016/j.radphyschem.2004.05.053

[70] Nikjoo H, O’Neill P, Wilson WE, Goodhead DT. Computational approach for determining the spectrum of DNA damage induced by ionizing radiation. Radiation Research. 2001;156(5 II):577–583. doi: 10.1667/0033-7587(2001)156[0577:cafdts]2.0.co;2 11604075

[71] Jenner TJ, DeLara CM, O’Neill P, Stevens DL. Induction and rejoining of DNA double-Strand breaks in v79-4 mammalian cells following *γ* and *α*irradiation. International Journal of Radiation Biology. 1993;64(3):265–273. doi: 10.1080/09553009314551421 8105005

[72] Frankenberg D, Brede HJ, Schrewe UJ, Steinmetz C, Frankenberg-Schwager M, Kasten G, et al. Induction of DNA double-strand breaks by 1H and 4He ions in primary human skin fibroblasts in the LET range of 8 to 124 keV/*μ*m. Radiation Research. 1999;151(5):540–549. doi: 10.2307/3580030 10319727

[73] Neary GJ, Horgan VJ, Bance DA, Stretch A. Further data on DNA Strand breakage by various radiation qualities. International Journal of Radiation Biology. 1972;22(6):525–537. doi: 10.1080/09553007214551431 4539380

[74] Morthekai P, Tiwari P, Murari MK, Singh P, Thakur B, Manoj MC, et al. Further investigations towards luminescence dating of diatoms. Radiation Measurements. 2022;156(June):106803. doi: 10.1016/j.radmeas.2022.106803

[75] Brown JE, Jones SR, Saxén R, Thørring H, Vives i Batlle J. Radiation doses to aquatic organisms from natural radionuclides. Journal of Radiological Protection. 2004;24(4 A). doi: 10.1088/0952-4746/24/4A/004 15700698

[76] Kumar A, Becker D, Adhikary A, Sevilla MD. Reaction of electrons with dna: Radiation damage to radiosensitization. International Journal of Molecular Sciences. 2019;20(16). doi: 10.3390/ijms20163998 31426385 PMC6720166

[77] Siefermann KR, Liu Y, Lugovoy E, Link O, Faubel M, Buck U, et al. Binding energies, lifetimes and implications of bulk and interface solvated electrons in water. Nature Chemistry. 2010;2(4):274–279. doi: 10.1038/nchem.580 21124507

[78] Lampe N, Karamitros M, Breton V, Brown JMC, Sakata D, Sarramia D, et al. Mechanistic DNA damage simulations in Geant4-DNA Part 2: Electron and proton damage in a bacterial cell. Physica Medica. 2018;48(November 2017):146–155. doi: 10.1016/j.ejmp.2017.12.008 29371062

[79] Krasovec M, Sanchez-Brosseau S, Piganeau G, Baer C. First Estimation of the Spontaneous Mutation Rate in Diatoms. Genome Biology and Evolution. 2019;11(7):1829–1837. doi: 10.1093/gbe/evz130 31218358 PMC6604790

