# Supplementary material for: Assessing radiation dosimetry for microorganisms in naturally radioactive mineral springs using GATE and Geant4-DNA Monte Carlo simulations
Source: PLoS One. 2023 Oct 12;18(10):e0292608. doi: 10.1371/journal.pone.0292608 (PMC10569590; doi:10.1371/journal.pone.0292608)
Supplement: Attachment — Submitted filename: Response_to_Reviewers.pdf [file pone.0292608.s001.pdf]

Dear editor,

We would like to express our gratitude for the interest expressed for our manuscript. We highly appreciate the relevance of all the comments made by the reviewers and your additional guidance to meet the journal's requirements.

Please find below our answers to the reviewer comments as well as a proposed revision of the manuscript. We hope this revised version will improve its quality, meet the journal's criteria, and merit publication.

Kind regards,

Kolovi Sofia on behalf of the authors

Journal Requirements:

When submitting your revision, we need you to address these additional requirements.

Requirement 1. Please ensure that your manuscript meets PLOS ONE's style requirements, including those for file naming. The PLOS ONE style templates can be found at https://journals.plos.org/plosone/s/file?id=wjVg/PLOSOne_formatting_sample_main_body.pdf and https://journals.plos.org/plosone/s/file?id=ba62/PLOSOne_formatting_sample_title_authors_affiliations.pdf

Response to Requirement 1: The manuscript has been revised according to the plos_latex_template_v3.6 (https://journals.plos.org/plosone/s/latex).

Requirement 2. In your Methods section, please provide additional information regarding the permits you obtained for the work. Please ensure you have included the full name of the authority that approved the field site access and, if no permits were required, a brief statement explaining why.

Response to Requirement 2: No authority permissions are required for the studied mineral springs since they belong to the public sector with no restrictions applied. In order to clarify this point, additional text was added in Line 100: "The springs are open for public use and no permit was required to access them and collect samples".

Requirement 3. Please note that PLOS ONE has specific guidelines on code sharing for submissions in which author-generated code underpins the findings in the manuscript. In these cases, all author-generated code must be made available without restrictions upon publication of the work.

Please review our guidelines at https://journals.plos.org/plosone/s/materials-and-software-sharing#loc-sharing-code and ensure that your code is shared in a way that follows best practice and facilitates reproducibility and reuse.

Response to Requirement 3: The code can be found in https://github.com/lpc-umr6533/tiramisu_simulation and it will be immediate available to the public once required by the journal.

Requirement 4. We note that the grant information you provided in the 'Funding Information' and 'Financial Disclosure' sections do not match.

When you resubmit, please ensure that you provide the correct grant numbers for the awards you received for your study in the 'Funding Information' section.

Response to Requirement 4: We modified the "Funding Information" section accordingly.

Requirement 5. Please expand the acronym "CNRS" (as indicated in your financial disclosure) so that it states the name of your funders in full.
This information should be included in your cover letter; we will change the online submission form on your behalf.

Response to Requirement 5: Centre National de la Recherche Scientifique (CNRS)

Requirement 6. Thank you for stating the following financial disclosure:

"This study was funded by "Prime80 CNRS", contract No 1083577"

Please state what role the funders took in the study. If the funders had no role, please state: "The funders had no role in study design, data collection and analysis, decision to publish, or preparation of the manuscript." If this statement is not correct you must amend it as needed.

Please include this amended Role of Funder statement in your cover letter; we will change the online submission form on your behalf.

Response to Requirement 6: The text in the financial disclosure has been modified as following: "The current study was performed under S. Kolovi's PhD funding received by the Centre National de la Recherche Scientifique (CNRS) as a "Prime80 CNRS" project with contract No 1083577. The funders had no role in study design, data collection and analysis, decision to publish, or preparation of the manuscript."

Requirement 7. In your Data Availability statement, you have not specified where the minimal data set underlying the results described in your manuscript can be found. PLOS defines a study's minimal data set as the underlying data used to reach the conclusions drawn in the manuscript and any additional data required to replicate the reported study findings in their entirety. All PLOS journals require that the minimal data set be made fully available. For more information about our data policy, please see http://journals.plos.org/plosone/s/data-availability.

Upon re-submitting your revised manuscript, please upload your study's minimal underlying data set as either Supporting Information files or to a stable, public repository and include the

relevant URLs, DOIs, or accession numbers within your revised cover letter. For a list of acceptable repositories, please see http://journals.plos.org/plosone/s/data-availability#loc-recommended-repositories. Any potentially identifying patient information must be fully anonymized.

Important: If there are ethical or legal restrictions to sharing your data publicly, please explain these restrictions in detail. Please see our guidelines for more information on what we consider unacceptable restrictions to publicly sharing data: http://journals.plos.org/plosone/s/data-availability#loc-unacceptable-data-access-restrictions. Note that it is not acceptable for the authors to be the sole named individuals responsible for ensuring data access.

We will update your Data Availability statement to reflect the information you provide in your cover letter.

Response to Requirement 7: We modified the Data Availability statement as it follows: "The simulation code is available on GitHub (https://github.com/lpc-umr6533/tiramisu_simulation). All relevant parameters and data are within the manuscript, allowing the reproducibility of the results."

Requirement 8. One of the noted authors is a group or consortium: TIRAMISU collaboration

In addition to naming the author group, please list the individual authors and affiliations within this group in the acknowledgments section of your manuscript. Please also indicate clearly a lead author for this group along with a contact email address.

Response to Requirement 8: The affiliations of the authors are added in the acknowledgements section. The lead author of the collaboration is among the co-authors of the article and his email address has been communicated accordingly below the authors list.

Requirement 9. Please review your reference list to ensure that it is complete and correct. If you have cited papers that have been retracted, please include the rationale for doing so in the manuscript text, or remove these references and replace them with relevant current references. Any changes to the reference list should be mentioned in the rebuttal letter that accompanies your revised manuscript. If you need to cite a retracted article, indicate the article's retracted status in the References list and also include a citation and full reference for the retraction notice.

Response to Requirement 9: One reference was removed because the article is still under revision and it has not been accepted yet (Line 68: Baker LA, et al.). Additional references were added, and the reference list was accordingly reviewed. Both the removed and added references are stated at the end of the rebuttal letter.

Response to Reviewer #1

Point 0 - The manuscript presents calculations of absorbed dose and dose rate to microorganisms living in mineral springs with high abundance of naturally occurring radioactive elements. The calculations are based on measured activity concentration and performed by available program code for Monte Carlo simulations. The manuscript is well written and structured but a few issues need to be addressed.

Response to Point 0 – We are very thankful for the interest expressed by the reviewer and the very useful comments which highlighted important aspects of our work. The points raised are addressed below.

Point 1, p.4 - Water was collected in beakers that were sealed to avoid leakage of Rn-222. It should be described how the sampling was performed to avoid exhalation of radon gas during the sampling.

Response to Point 1 - Water sampling was performed according to ISO 5667-1 standard for water sampling and ISO 5667-3 standard concerning water sample handling and preservation for radiochemical analysis. We used Marinelli style gas analysis containers from NUVIA Instruments GMBH that are specifically designed for γ-spectroscopy analyses of gases including radon. We added this information in the text:

Lines 111 - 113: "Water was collected according to ISO 5567-1 and ISO 5567-3 standards in 1 L Marinelli style gas analysis containers (NUVIA Instruments GMBH) designed for γ-spectroscopic analysis. The containers were sealed to avoid any ^{222}Rn leakage."

Point 2, p.4 - It is not obvious if the contribution to absorbed dose from all alpha emitters in the uranium and thorium decay chains is considered, or if only the contribution from Rn-222 and Ra-226 is considered. This is important to discuss in the discussion part, especially when making comparisons with the ERICA tool.
Table 2: Neither the uranium nor the thorium decay chains seem to be in equilibrium. Instead there seems to be an enrichment of Ra at most of the studied sites. The reason for this ought to be explained. The degree of disequilibrium will also affect the absorbed dose to organisms in these environments, if all alpha emitters are considered.

Response to Point 2: We would like to thank the reviewer for this highly important remark. It is now clarified in the manuscript that only the contribution of ^{222}Rn and ^{226}Ra is considered. Indeed, active hydrothermal circulation affects numerous areas of Massif Central. A common feature of several CO_2-rich geothermal systems is a high radium content acquired during the ascent of water from the deep reservoir through the crystalline bedrock. High radium solubility in the mineral waters can be related to the major ion composition [21]. The following text was added:

Lines 127 - 133: "A common feature of several CO_2-rich geothermal systems is a high radium content acquired during the ascent of water from the deep reservoir through the crystalline bedrock. High radium solubility in the mineral waters can be related to their major ion

composition [21]. As a consequence, the sediments and deposits (travertine) precipitated from the waters display a disequilibrium in the uranium and thorium decay chains."

The text between Lines 197 - 199 was also modified accordingly: "Following the reduced levels of activities measured for ^{238}U, ^{228}Ra and ^{228}Th (see Table 2) in comparison to ^{222}Rn and ^{226}Ra, as well as the disequilibrium of the decay chains observed in the sediments, we simulated only α-particles emitted by the latter without considering their daughter nuclei."

In the Discussion part, text was added in Lines 357 – 359: "The simulations were performed separately for the α-particles of ^{222}Rn and ^{226}Ra, excluding their daughter nuclei."

Lines 393 – 396: "The disequilibrium observed in the ^{238}U and ^{232}Th decay chains confirms that the dose rate calculations should not be performed under the equilibrium hypothesis but rather according to the measured activity concentrations, as adopted by ERICA."

Response to Reviewer #2

Point 0 - I have had an opportunity to review an interesting submission with a title "Simulation of the radiation exposure of microorganisms living in naturally radioactive mineral springs using GATE and Geant4-DNA Monte Carlo simulation tools" by Sofia Kolovi et al.
In general, the paper is well thought out and well written, in a concise and understandable English. Despite that, I have found few things in the running text, mentioned further below in my comments, which I think needs attention.
In the article the authors have taken samples from five different natural mineral springs in France, and performed radiological characterisation of these springs. With the calculated activity concentration values, they then tried to mimic the conditions within the springs in a Geant4 model at three positions – sediment position, mixed position and water position, replicating radiological conditions at these positions in the springs. Then they created a semi-detailed diatom algae model out of spheres of water, and tried to estimate the amount of single and double strand breaks in the DNA of the diatom algae.
The results are interesting, as they show that the number of double strand brakes, which is indirectly correlated with the number of mutations of the DNA, can be expected to be high in such mineral spring conditions – particularly spring five – than as for other microorganisms in normal radiation background environments. It would be interesting to see this applied to help solving the human low-dose radiation response problem in radiation protection by trying similar studies for human tissues.
I recommend a minor revision.

Response to Point 0 – We would like to thank the reviewer for their interest and the encouraging review. The comments were all well taken into serious consideration and respective modifications in the text were applied.

Below you will find my comments to this manuscript:
Point 1, Line 151 - I think it would be helpful to at least mention what is considered to be the

deformation, not just leaving a reference to another paper. I think, lightly incorporating a brief definition of a deformation would be very helpful for the readers.

Response to Point 1 – The corresponding text has been supplemented by the description of the deformations considered in reference [35] as shown below:

Lines 161 - 164: "The same species are observed at spring 5 (Chateldon) where deformation rates above 25%, denoted by abnormally shaped frustules, have been observed on *Planothidium frequentissimum* [35]. Diatom deformations or else stated "teratological formations" are abnormalities on their morphology, mainly on the valve shape and structural characteristics which result in deformed frustules."

Point 2, Line 188 - "were emitted isotropically" – I would say being a bit more detailed than isotropically would be very helpful for the clarity of the text. Something along the lines of "the modelled radioactive nuclei were distributed randomly across material X and with the directions random in the solid angle of X π…" would be way more clearer for the reader.

Response to Point 2 - The suggestion has been implemented in the text as it follows:
Lines 202 – 205: "For each radionuclide, the α-particles were emitted isotropically (4π solid angle) from the spherical volume surrounding the microorganism where their emission point was randomly distributed."

Point 3 - In Table 4, a list of energies of α-particles is provided. Detail is lacking regarding how they were simulated and calculated. In the running text it is mentioned that the total energy deposited was counted, but later in results, the results for 226Ra and 222Rn are separated. Could you please clarify this a bit more?

Response to Point 3 - References[1,2] were added in Table 4 legend pointing to the databases from which the α-particles energies were retrieved. Additional text was inserted in order to clarify that the two radionuclides were simulated separately:

Line 205: "Separate simulations were performed for ^{222}Rn and ^{226}Ra."
Lines 223 - 224: "We ran separate simulations with 10E+08 α-particles for each radionuclide and repeated them 10 times to evaluate the statistical fluctuations (kept below 1%)."

Regarding the "total energy deposited", indeed, the energy deposited by each radionuclide was recorded and then, the total energy deposited was also calculated. We modified the text accordingly:

Lines 218 - 220: "Then, the energy deposited to the microorganism for each radionuclide was recorded, the contributions of the dominant physical processes were identified, and the total energy depositions were calculated."

Point 4, Line 269 - Am I misunderstanding something, or is "ion ionisation" a typo? Can you really ionise an ion?

Response to Point 4 – We would like to thank the reviewer for this very critical remark. Indeed, we kept the terminology from Geant4 concerning the mechanisms describing energy depositions. The alpha particle is classified as an ion in the MC code since it is a double-ionised

He atom. When the alpha particle ionizes the matter, the process responsible for the energy deposited is “IonIonisation”.
As long as the alpha particle is able to produce secondary electrons able to further ionise the medium (called δ-rays), the energy depositions of these secondary electrons are denoted as “electron ionisations”. In order to clarify this for the reader, the following text was added:

Lines 289 – 291: “Ion ionisation refers to the ionisations caused directly from the α-particle while the electron ionisation refers to the ionisations caused by sufficiently energetic secondary electrons (δ-rays).”

Point 6 - In Table 10, the values displayed in the parentheses are for diatoms with frustules. This needs to be mentioned explicitly above or below the table. Otherwise it can get confusing for the reader, and it takes unnecessary time to work this out.

Response to Point 6 – We thank the reviewer for highlighting this potential point of confusion for the reader. We added the following part in the legend of Table 10:

“Table 10. Mean specific energy (SE) rates per nucleosome (μGy/h) and number of SSBs, DSBs (when considering frustule, values are provided in the parenthesis)”

Point 7, Line 372 - It would be nice if the “solvations of secondary electrons” would be very briefly explained to avoid confusion, as the immediate-previous context of this phrase within the running text discusses physics of radiation interaction with matter.

Response to Point 7 – The following text was added:

Line 405: “Solvated electrons (free electrons in liquid water) known to play an important role in the damaging effects of DNA [73,74] to…”

Point 8, Figure 6 – There is basically no discussion regarding the comparison of the results of the article to the other studies, apart from saying that the values are in “good agreement”. I think, a bit more thorough comparison – with at least one of the other studies – would be very beneficial to further strengthening the paper. Additionally, it would be very nice to see how the error bars look on all of the SpointProb graphs.

Response to Point 8, Figure 6 – We understand the concern raised by the plot. Its discussion is detailed later in the paper (lines 415 - 421). We modified the text as it follows:

Lines 322 - 327: “As it is shown, our predicted values are in general agreement with the experimental and other simulation results using different MC codes. The overprediction of DSBs when using the suggested values of 16% [53] and 20% [49] smooths when lowering the value. 12% offers a good agreement with H. Moeini et al. [64] but we finally chose SpointProb equal to 8% due to the better agreement with the experimental results, too.”

We hope that this modification fits well with the Discussion lines 415 - 421, where we provide the full explanation of the impact of the modification of SPointProb values on the results.

In order to evaluate the error bars, only due to the statistics, in Fig 6, we ran the simulation multiple times and we achieved a relative uncertainty below 0.1% (too small to be visible in the graph). For clarification, the following text was added:

Line 275: "We ran the simulation multiple times, achieving a relative uncertainty below 0.1%."

ADDITIONAL REFERENCES

1. Singh, S., Jain, A. K. & Tuli, J. K. Nuclear Data Sheets for A = 222. *Nucl. Data Sheets* **112**, 2851–2886 (2011).
2. Singh, B. *et al.* Nuclear Data Sheets for A=218. *Nucl. Data Sheets* **160**, 405–471 (2019).

REMOVED REFERENCES
From line 68:

Baker L.A., Beauger A., Kolovi S., Voldoire O., Allain E., Breton V., Chardon P., Miallier D., Bailly C., Montavon G., Bouchez A., Rimet F., Chardon C., Vasselon V., Ector L., Wetzel C.E., Biron D.G. Diatom DNA metabarcoding to assess the effect of natural radioactivity in mineral springs on genetic variants of benthic diatoms communities. Submitted to Science of the total environment. September 2002.
