# Supplementary material for: Assessing radiation dosimetry for microorganisms in naturally radioactive mineral springs using GATE and Geant4-DNA Monte Carlo simulations
Source: PLoS One. 2023 Oct 12;18(10):e0292608. doi: 10.1371/journal.pone.0292608 (PMC10569590; doi:10.1371/journal.pone.0292608)
Supplement: Attachment — Submitted filename: Response_to_Reviewers.pdf [file pone.0292608.s002.pdf]

Dear editor,

We would like to express our gratitude for the interest expressed for our manuscript. We highly appreciate the relevance of all the comments made by the reviewers.

Please find below our answers to the reviewer comments as well as a proposed revision of the manuscript. We performed two corrections due to incorrect data processing in the manuscript regarding the comparison of our results with the publication of Krasovec et al. as indicated below:

Lines 440 - 444: "Recently, experiments to evaluate the spontaneous mutation rate in the model diatom Phaeodactylum tricornutum were conducted for the first time revealing a total nuclear mutation rate of about 5.00E-10 per nucleotide per generation (accounting for base substitution and insertion-deletion mutation rates)"

**by**

"Recently, experiments to evaluate the spontaneous mutation rate in the model diatom Phaeodactylum tricornutum were conducted for the first time revealing a **total spontaneous nuclear mutation rate per generation of approximately 1.29E−02** (accounting for base substitution and insertion-deletion mutation rates)"

Lines 445 - 448: "When comparing these numbers to the rate of radiation induced SSBs and DSBs deduced from Table 10, respectively about 0.174E-10 and 0.04E-10 per nucleotide per day, for the benthic mixture, we observe that radiation induced mutations contribute to an accountable mutational pressure."

**by**

"When comparing these numbers to the rate of radiation induced SSBs and DSBs deduced from Table 10, **respectively 4.70E-04 and 1.11E-04 per generation day** for the benthic mixture, we observe that radiation induced mutations **could** contribute to an accountable mutational pressure."

We hope this revised version will improve its quality, meet the journal's criteria, and merit publication.

Kind regards,

Kolovi Sofia on behalf of the authors

## *Journal Requirements:*

**Requirement 1.** Please review your reference list to ensure that it is complete and correct. If you have cited papers that have been retracted, please include the rationale for doing so in the manuscript text, or remove these references and replace them with relevant current references. Any changes to the reference list should be mentioned in the rebuttal letter that accompanies your revised manuscript. If you need to cite a retracted article, indicate the article's retracted status in the References list and also include a citation and full reference for the retraction notice.

Response to Requirement 1: Two references were added in compliance with extra material asked by the reviewers.

- d-maps. Available from: https://d-maps.com/index.php?lang=en
- NIST National Institut of Standards and Technology Materials Database. Available from: https://materialsdata.nist.gov/.

## *Response to Reviewer #2*

**Point 0 -** I am happy in the way the comments have been addressed, and the text of manuscript altered. I have no further comments.

Response to Point 0 – We are grateful that our revision met the reviewer's expectations.

## *Response to Reviewer #3*

**Point 0 -** Overall: This is an extremely interesting topic. This multi-disciplinary study appears to have been planned and executed in accordance with standards and accepted experimental protocols.

Introduction: This section is well written with a clean layout. Readers will appreciate the step-by-step introduction of the finer points of the experiment motivation.

Response to Point 0 – We are very thankful for the interest expressed by the reviewer and the useful comments. The points raised are addressed below.

**Point 1, Line 87 -** Briefly define and describe the DBSCAN algorithm.

Response to Point 1 – The following text was added in lines **87 – 92**:

**DBSCAN (Density Based Spatial Clustering of Applications with Noise). For the calculation of DNA damage, DBSCAN takes into account the distribution of deposited energy induced by ionising radiation (α-particles in our case) in micrometric geometries and a damage probability function which depends on the total deposited energy.**

Materials and methods: I appreciated the detailed description of the sampling methods, protocols, and standards followed. The Geant4 geometry was modeled appropriately for the scenarios and described effectively.

**Point 2, Table 1 -** It might be useful to show a map showing the sample locations of the springs amongst the macroscopic bodies of water in the area for readers unfamiliar with the location.

Response to Point 2 – **A map (Figure 1) was added to the main body of the manuscript along with images of three of the mineral springs.**

**Point 3, Table 2** - I think +/- symbols may be missing in the numeric columns in this PDF version?

Response to Point 3 – **Table 2 was modified accordingly.**

**Point 4, Line 235** - Please explain why pure water is the appropriate material choice for the nucleosome cylinders in the model? I am left wondering why a mixture of DNA elements were not included at their respective average abundances within the nucleosome volumes.

Response to Point 4 – **This very interesting point is one of the major research topics of Geant4-DNA collaboration. Currently, the cross sections available in Geant4-DNA are validated for water (as defined by NIST). The following text was added in:**

lines **217 – 218: G4_WATER was used as material in all cases.**

lines **243 – 244: All G4-DNA models are validated and available for water (G4 WATER as defined in NIST) which is used as a surrogate to the biological medium.**

Results:
**Point 5, Table 9 -** The use of the values in parentheses was a bit confusing at first but could be deciphered from the text above with some additional effort. Recommend adding similar clarification as is included in the caption of Table 10 ("when considering frustule, values are provided in the parenthesis") to the caption of Table 9 as well. It is curious that the mean and maximum energies were reduced but the mean deposited energy was not? Perhaps include further explanation on this finding.

Response to Point 5 – **We appreciate a lot this concern. For clarification purposes, we added the indicated text in the captions of Tables 7, 8 and 9.**

**(“when considering frustule, values are provided in the parentheses”)**

**Considering the non-obvious decrease of the mean deposited energy, we added 2 decimals in the respective values of Table 9. We would like to specify that in Table 9, we are providing only the mean and maximum kinetic energies, not the whole energy spectrum. As a result, conclusions about the relation between the reduced kinetic energy of the particles reaching the nucleus and the energy deposition at the nucleus cannot be straightforward drawn. The following text was also added in:**

lines **318 – 319: We also notice that the frustule has a very limited impact on the deposited energy.**

**Point 6, Figure 5 -** Define axis units in text or caption

Response to Point 6 – **The unit of y-axis ($Gy^{-1}$) was added in the captions of Figures 4, 5 and 6.**

Discussion/Conclusion:

**Point 7, Line 300-301,356-357,468 -** Line 300-301 and Figure 2 show a 10% decrease in dose rate (10.8 to 9.7 uGy/h) with frustule. Table 8 shows only one absolute dose decrease with frustule of over 20%, while the other absolute dose and dose rate deltas are between 8% and 17%. Where is the data supporting 20% dose decrease with frustule listed in lines 356-357 and 468? Please augment discussion to clarify.

Response to Point 7 – **We would like to thank the reviewer for pointing out this discrepancy between the results shown in the manuscript and the discussion part. First, we removed the respective text from line 365 where we discuss about the 20% decrease in the number of α-particles reaching the diatoms.**

**Secondly, we would like to justify our calculations. In Table 8, the absolute doses in the benthic mixture are decreased due to the presence of the frustule by 22% for $^{226}$Ra and by 17.1% for $^{222}$Rn. As a result, we indicate an average 20% decrease of absolute doses.**

**For clarification, we modified the text in line 478 as follows:**

line **478: “We showed that the main contributor to the doses is $^{226}$Ra and that the frustule is responsible for an average 20% absolute dose decrease”**

## *Response to Reviewer #4*

**Point 0 -** This work is the first endeavor to utilize multiscale Monte-Carlo simulations to evaluate the radiation exposure of microorganisms existing in a naturally radioactive environment from α-emitters including 226Ra and 222Rn. It is innovative and provides very valuable research results and experimental data and has the potential to be employed in upcoming radioecological investigations that intend to evaluate both radiation dose rates and potential DNA damages to aquatic microorganisms and extend to other radioisotopes. The manuscript is well-written.

Response to Point 0 – We would like to thank the reviewer for their interest and the very encouraging review.

**Point 1, Lines 66 – 69 -** "An exceptional abundance of deformations in the most radioactive springs in Auvergne has been recently revealed, initiating studies of the effects of natural radioactivity on benthic diatom communities in 16 mineral springs of the area."
Out of 16 mineral springs, 5 were chosen. What is the reason for choosing these 5 springs? Is it according to the degree of activity concentrations of 222Rn in water?

Response to Point 1 – **We would like to thank the reviewer for their high interest in our research. These 5 locations were among the first to be identified and triggered our interest during the initial surveys in 2017. The research has been ongoing ever since, with valuable results presented in 2019 by Milan et al. which have led to more extensive studies in the region as presented by Baker et al. in 2022.**

**Point 2, Table 2 -** Why the sampling date for Chateldon mineral spring is March 2017, not a specific date as those for other mineral spring? Is it because that the measured mass activities of radionuclides for Chateldon mineral spring is an average of multiple measurements in March?

Response to Point 2 – **We would like to apologize for omitting the specific date of the sediment sampling in March 2017 at Chateldon. The sampling was performed on 01/03/2017 and it does not concern multiple measurements. Table 2 is now modified accordingly.**

.
