# Supplementary material for: Assessing radiation dosimetry for microorganisms in naturally radioactive mineral springs using GATE and Geant4-DNA Monte Carlo simulations
Source: PLoS One. 2023 Oct 12;18(10):e0292608. doi: 10.1371/journal.pone.0292608 (PMC10569590; doi:10.1371/journal.pone.0292608)
Supplement: Attachment — Submitted filename: ResponseToReviewers.pdf [file pone.0292608.s004.pdf]

Dear editor,

We are grateful for having stood up to the expectations of the reviewers and we highly appreciate your interest in our work and the very productive comments. Please find below our corresponding responses, as well, as a proposed revised version of the manuscript.

We hope that this revision will improve the quality of the manuscript, meet the journal's criteria, and merit publication.

Kind regards,

Kolovi Sofia on behalf of the authors

Academic editor comments:

Point 1, Title - *Too concise. Double usage of "simulation" and it is too general. I believe titling an article would help the readers for their research interest and helping the keyword searching, plus increase the citation. The scope of work is in the dosimetry field. I would like to suggest the author to change the title to more technical one:*

"Dosimetric technique for exposed microorganism to naturally radioactive mineral springs using GATE and Geant4-DNA Monte Carlo simulation".

Or;

"Assessing Radiation Dosimetry for Microorganisms in Naturally Radioactive Mineral Springs using GATE and Geant4-DNA Monte Carlo Simulations".

Response to Point 1 – **We fully embrace the need of modifying the title. The title was replaced by:**

"Assessing Radiation Dosimetry for Microorganisms in Naturally Radioactive Mineral Springs using GATE and Geant4-DNA Monte Carlo Simulations"

Point 2, Abstract - *The important part to give reader insights and highlighted finding of you work. Please include:*

-radioactivity levels of Ra-226 & Rn-222 of the spring

- a bit of technique used in this work (2-3 lines), what tools? Radiation source specs, any reference phantom, water sphere, simplified spherical geometry? Radiation transport physics treatment? Geant4 electromagnetic physics list option 4?

-your results (simulated dose rates), DNA damage counts/indicator and the threshold levels (ICRP or IAEA)?

- Revise this into separable text and please specify your investigated biota/species (eukaryotic photosynthetic 57 unicellular microalgae).

"We focus on the impact of the dominant radioelements, ^{226}Ra in the sediments and ^{222}Rn dissolved in the water of the mineral springs, and on diatoms, microalgae displaying an exceptional abundance of teratological forms in the most radioactive springs studied in Auvergne".

-The conclusion is quite brief, any implication on the investigated species in future based on ICRP Publication 108 or related to Reference Animal in Publication 108.

Response to Point 2 – **The abstract was revised according to the suggestions. We would like, nevertheless, to provide some clarifications:**

- **This work involves the collection of experimental data of 5 mineral springs. Nonetheless, the results focus on and are normalized to the reference values of 1000 Bq/L 222-Rn and 30 Bq/g 226-Ra, as stated in lines 141-144 of the manuscript, which do not explicit correspond to one specific mineral spring.**
- **Concerning the simulated microorganism, no correspondence to the Reference Animals and Plants of ICRP Publication 108 is feasible. There is still a lot of on-going work for diatoms, including their irradiation in controlled environments.**

The following modifications were performed:

"Mineral springs in Massif Central, France can be characterized by higher levels of natural radioactivity in comparison to the background. The biota in these waters is constantly under radiation exposure mainly from the α-emitters of the natural decay chains, **with 226Ra in sediments ranging from 21 Bq/g to 43 Bq/g and 222Rn activity concentrations in water up to 4600 Bq/L**. This study **couples** ~~proposes~~ ~~to apply~~ for the first time micro- and nanodosimetric approaches **to radioecology by** ~~and tools~~ **combining GATE and Geant4-DNA** to ~~evaluate~~ **assess** the dose **rates and DNA damages** ~~received by~~ to microorganisms living in **these** naturally radioactive ecosystems. ~~We focus on the impact of the dominant radioelements, 226-Ra in the sediments and 222-Rn dissolved in the water of the mineral~~

~~springs, and on diatoms, microalgae displaying an exceptional abundance of teratological forms in the most radioactive springs studied in Auvergne~~. **It focuses on unicellular eukaryotic microalgae (diatoms) which display an exceptional abundance of teratological forms in the most radioactive mineral springs in Auvergne. Using spherical geometries for the microorganisms and based on γ-spectrometric analyses, we evaluate the impact of the external exposure to 1000 Bq/L 222-Rn dissolved in the water and 30 Bq/g 226-Ra in the sediments.** Our results show that the **external** dose rates **for diatoms** are significant **(9.7 µGy/h) and** comparable to the threshold **(10 µGy/h)** for the protection of the ecosystems **suggested by the literature.** ~~They also offer a first evaluation of the radiation induced DNA damage on these species~~. **In a first attempt of simulating the radiation induced DNA damage on this species, the rate of DNA Double Strand Breaks per day is estimated to 1.11E-04.** Our study confirms the significant mutational pressure from natural radioactivity to which microbial biodiversity has been exposed since Earth origin in hydrothermal springs."

Point 3, Introduction, Paragraph 3 - *please remove. "From the need of years lasting ……….unique". The texts given here doesn't help reader gain insights of the purpose of the study. Please include the importance or the necessity of dose assessment for microbes/microorganisms. No solid technique for micro biotas dose assessment? For advancement in scientific knowledge of radioecology field?*

Response to Point 3 – **The indicated text has been removed. Additional text has been added in line 25 and line 40 in order to further highlight the importance and the necessity for micro biota dose assessments.**

Line 25: …**following the ICRP (International Commission on Radiological Protection) approach**…

Line 40: **Aiming to cover the needs of microscale radioecology,….**

Point 4, Line 52-53 – *how high? Please include the activity levels.*

Response to Point 4 – **Additional text was introduced as follows:**

Lines 54 - 55: ...are found in high concentrations in the sediments **(up to 31 Bq/g 226-Ra)** and waters **(up to 4600 Bq/L 222-Rn)** of the mineral springs in Auvergne [35].

Point 5, Line 89 - *chronic exposure to ionizing radiation.*

Response to Point 5 - **The text was modified accordingly in Line 91.**

Point 6, Line 118 - *please give specifically how many hours before gamma counting? To allow secular equilibrium? Please elaborate.*

Response to Point 6 – **γ- spectroscopy for 222-Rn dissolved in water was performed within the first 3 hours of sampling in order to allow for an integral measurement of the total radioactivity due to 214-Pb. This radon is not at secular equilibrium with 226-Ra in water. A second measurement can be done one month after sampling to measure 222-Rn sustained by 226-Ra in water that amounts to less than a few Bq/L.**

The respective text was modified as follows:

Line 120: 222-Rn activity concentrations were measured within **the first 3** hours after sampling …

Point 7, Line 178 - *Justify why silicate shell need to model for diatom? My apology for mislook on any text that describe the anatomy of the studied diatom in section line 149 - 172, is there any elemental composition (SEM EDX) of these to justify the MC simulation diatoms phantoms?*

Response to Point 7: **Diatoms are the only known microorganisms with a rigid silicate frustule. Given the general morphology of diatoms described in lines 63 – 65, as well as, the highlighted importance of the frustule in lines 66 – 70 and lines 167 – 170, we assumed that the when the reader reaches line 180, he/she is already aware that diatoms are characterized by a silicate exoskeleton which is not encountered in any other microorganism. To improve the readability, we added the following text for clarification:**

Line 181: …around the microorganism, **representing their frustule (rigid exoskeleton).**

Point 8, Lines 200 – 203: *The text cannot simply justify because of factor of low activity of U, Ra-228, Th in water spring for this study to choose Rn and Ra-226 only. There must a solid reason why this study chooses Rn and Ra over others. Line 49-53 already highlighted it.*

Response to Point 8: **We thank the editor for this comment. Indeed, the intention of this text was to justify why the daughter nuclei of 226-Ra and 222-Rn were not simulated. Indeed, this study focuses on the dose rate to microorganisms coming from the radioelements that have been measured experimentally to have the highest activity in the sediments and waters of Auvergne springs. These radioelements also decay and their daughters contribute an additional radiation dose to the microorganisms. In a first step, this contribution was not computed using Monte-Carlo simulations because the chemical behaviour of these radionuclides and therefore their location in the vicinity of the diatoms is not known. As a result, the text was modified accordingly:**

Lines 202 – 212: "~~Following the reduced levels of activities measured for 238-U, 228-Ra and 228-Th (see Table 2) , in comparison to 222-Rn and 226-Ra$~~,as ~~well as the disequilibrium of the decay chains observed in the sediments~~, <u>**This study focuses on the dose rate to microorganisms coming from the radioelements that have been measured experimentally to have the highest activity in the sediments and waters of Auvergne spring.**</u> <u>**As a consequence,**</u> we simulated only the α-particles emitted <u>**directly**</u> by 222-Rn and 226-Ra ~~without considering their daughter nuclei~~ (see Table 4). <u>**These radioelements also decay and their daughters, especially the α-emitters contribute an additional radiation dose to the microorganisms. In a first step, this contribution was not computed using Monte-Carlo simulations because the chemical behaviour of these radionuclides and therefore their location in the vicinity of the diatoms is not known.**</u>

Point 9, Line 214 - *an approximation is needed to extend the elaboration on the approach for radiation transport treatment. The question is; what is secondary electron energy range (control parameter) considered here for production cuts that stop tracking the particle further in Geant4 simulation. For smaller organisms, I believe the small energy contribution are important. Otherwise just state the approximation/simplification for physics treatment. Please elaborate the association of gammas? Because line 202 stated the consideration of alpha-particle only. Are they ancillary interaction of the alpha, please clarify.*

Response to Point 9: **The application of productions cuts is used to avoid the tracking of very low energy secondary particles which is computational expensive. Various cuts were preliminary tested in order to evaluate the impact on the dose rates before concluding in the values presented in this manuscript. Thus, the following text was added to further clarify the use of the suggested production cuts:**

Lines 223 – 225: The production cuts applied to secondary electrons and gammas, <u>**which are produced due to the interactions of the α-particles with the matter**</u>, <u>**were investigated in preliminary simulations,**</u> and chosen to be 2 orders of magnitude less than the size of the radius of the simulated volumes:…

Line 227: …<u>**,corresponding thus to the lowest cut-off energy available in GATE (250 eV).**</u>

Point 10, Line 353 - *revise accordingly as commented in line 200-203.*

Response to Point 10: **We replaced the text "We chose….in five different mineral springs" in Lines 364-366 with the following:**

Lines 366 - 372: <u>**We simulated separately the α-particles of 222-Rn in the water column and 226-Ra in the sediments of mineral springs in Auvergne, excluding their daughter nuclei in a first stage. Indeed, the contribution coming from the radioelements, especially the α-emitters in the decay chain of 226-Ra and 220-Rn depends on their location in the vicinity of the diatoms. Additional data are needed to understand their chemical behaviour and therefore their contribution to the dose rate to the microorganisms.**</u>

Point 11, Lines 360 – 362: *The statement of "external radiation exposure" simulation should be transferred in intro as well as in the abstract as it presents the scope of this study.*

Response to Point 11: **The following text was added in order to highlight the simulation of external radiation exposure:**

Abstract: … **we evaluate the impact of the external exposure to** …. Our results show that the **external** dose rates….

Introduction, Line 41: …for modelling the **external** radiation exposure and …

Point 12, Conclusion - *please conclude your work not summarizing it. You can directly point out on that average or range of absorbed dose received by the microorganism based on 3 simulation scenarios in 2-3 lines and conclude that the dose is compatible to threshold limit by ERICA (please use their original recommended levels by IAEA or ICRP), and conclude that the microorganism extremely exposed to DNA damages based on computed mutation rate or DSB or SSB.*

Response to Point 12: **The conclusion was modified according to the suggestions. Part of the text was moved to the Discussion as follows:**

Lines 455 – 458: With our work, we show that the computed mutation rate (4.7E-04 DSB/day) for the diatoms in the studied mineral springs is 4 orders of magnitude higher than for bacteria exposed solely to natural background radiation (2.8E-08 DSB/day) as calculated by Lampe et al. [78]

The modifications in the Conclusion are:

~~Five mineral springs in Auvergne, Massif Central, France were sampled and analysed using γ-spectrometry. Activity concentrations of 222-Rn in water and 226-Ra in sediments varied between 14 - 4600 Bq/L and 21 - 43 Bq/g, respectively. This work is, to our knowledge, the first attempt of a multiscale Monte-Carlo simulation of the radiation exposure of microorganisms living in a naturally radioactive ecosystem. It builds upon the experimental measurements of the radiological content of mineral springs and Monte-Carlo simulation tools, GATE and Geant4-DNA, that have been designed for biomedical applications. We focused on 222-Rn and 226-Ra α-emitters with a mean energy of 5 MeV, and diatoms, microalgae which inhabit these ecosystems. The microorganism and its nucleus was modelled as a water sphere of 10 µm and 0.5 µm radius, respectively, while the silicate frustule was considered of 2 µm width distinguishing the diatom from any other microorganism.~~ **In this study**, **we focused on the simulation of the external radiation exposure of microorganisms and diatoms living in naturally radioactive aquatic ecosystems.** Three different environmental compositions were simulated **corresponding to the ecosystems of mineral springs**: dry sediments (0\% porosity) containing only 30 Bq/g 226-Ra, water (100\% porosity) containing only 1000 Bq/L 222-Rn and a benthic mixture of both (90\% porosity) representing realistic conditions. In the benthic mixture, the diatom is exposed to 9.7 µGy/h due to 226-Ra and 222-Rn, a dose rate which is comparable with the threshold (10 µGy/h) for the protection of the ecosystems suggested by ERICA risk assessment tool. ~~We showed that the main contributor to the doses is $\Ra$ and that the frustule is responsible for an average 20\% absolute dose decrease.~~ ~~Using the DBSCAN clustering algorithm, we predicted 0.16 SSB/Gy/Mbp for diatoms (frustule) and 0.15 SSB/Gy/Mbp microorganisms (without frustule),~~

as well as, 0.03 DSB/Gy/Mbp for both organisms, showing that diatoms are not considerably protected from ionizing radiation due to their frustule. **We evaluated that the frustule does not considerably protect diatoms from ionizing radiation.** With our work, we show that the mutation rate (4.7E-04 DSB/day) for the diatoms in the studied mineral springs is 4 orders of magnitude higher than for bacteria exposed solely to natural background radiation (2.8E-08 DSB/day) as calculated by Lampe et al.}[78]. **Based on our computed DSB, we show that the microorganisms are extremely exposed to DNA damages due to the chronic exposure to ionising radiation in the radioactive mineral springs**.These results contribute to a better understanding of the radiation stress that microorganisms, chronically exposed to natural radioactivity, encounter. By demonstrating the coupling of experimental measurements with Monte Carlo simulations for two radionuclides, this work can be implemented in future radioecological studies wishing to estimate not only dose rates but also potential DNA damages on aquatic microorganisms and extend to other radioisotopes.
