# Supplementary material for: Assessing radiation dosimetry for microorganisms in naturally radioactive mineral springs using GATE and Geant4-DNA Monte Carlo simulations
Source: PLoS One. 2023 Oct 12;18(10):e0292608. doi: 10.1371/journal.pone.0292608 (PMC10569590; doi:10.1371/journal.pone.0292608)
Supplement: Attachment — Submitted filename: reviewer_comments_ PONE-D-22-32535R2.docx [file pone.0292608.s003.docx]

Academic editor comments:

Title – Too concise. Double usage of “simulation” and it is too general. I believe titling an article would help the readers for their research interest and helping the keyword searching, plus increase the citation. The scope of work is in the dosimetry field. I would like to suggest the author to change the title to more technical one:

“Dosimetric technique for exposed microorganism to naturally radioactive mineral springs using GATE and Geant4-DNA Monte Carlo simulation”.

Or;

“Assessing Radiation Dosimetry for Microorganisms in Naturally Radioactive Mineral Springs using GATE and Geant4-DNA Monte Carlo Simulations”.

Abstract – The important part to give reader insights and highlighted finding of you work. Please include:

-radioactivity levels of Ra-226 & Rn-222 of the spring.

- a bit of technique used in this work (2-3 lines), what tools? Radiation source specs, any reference phantom, water sphere, simplified spherical geometry? Radiation transport physics treatment? Geant4 electromagnetic physics list option 4?

-your results (simulated dose rates), DNA damage counts/indicator and the threshold levels (ICRP or IAEA)?

- Revise this into separable text and please specify your investigated biota/species (eukaryotic photosynthetic 57 unicellular microalgae).

“We focus on the impact of the dominant radioelements, ^226^Ra in the sediments and ^222^Rn dissolved in the water of the mineral springs, and on diatoms, microalgae displaying an exceptional abundance of teratological forms in the most radioactive springs studied in Auvergne”.

-The conclusion is quite brief, any implication on the investigated species in future based on ICRP Publication 108 or related to Reference Animal in Publication 108.

Introduction, para 3 – please remove. “From the need of years-lasting………………….unique”. The texts given here doesn’t help reader gain insights of the purpose of the study. Please include the importance or the necessity of dose assessment for microbes/microorganisms. No solid technique for micro biotas dose assessment? For advancement in scientific knowledge of radioecology field?.

Line 52-53 – how high? Please include the activity levels.

Line 89 – chronic exposure to ionizing radiation.

Line 118 – please give specifically how many hours before gamma counting? To allow secular equilibrium? Please elaborate.

Line 178 – Justify why silicate shell need to model for diatom? My apology for mislook on any text that describe the anatomy of the studied diatom in section line 149 - 172, is there any elemental composition (SEM EDX) of these to justify the MC simulation diatoms phantoms?

Line 200-203 – The text cannot simply justify because of factor of low activity of U,Ra-228, Th in water spring for this study to choose Rn and Ra-226 only. There must a solid reason why this study chooses Rn and Ra over others. Line 49-53 already highlighted it.

Line 214 – an approximation is needed to extend the elaboration on the approach for radiation transport treatment. The question is; what is secondary electron energy range (control parameter) considered here for production cuts that stop tracking the particle further in Geant4 simulation. For smaller organisms, I believe the small energy contribution are important. Otherwise just state the approximation/simplification for physics treatment. Please elaborate the association of gammas? Because line 202 stated the consideration of alpha-particle only. Are they ancillary interaction of the alpha, please clarify.

Line 353 – revise accordingly as commented in line 200-203.

Line 360 – 362 - The statement of “external radiation exposure” simulation should be transferred in intro as well as in the abstract as it present the scope of this study.

Line 363 – redundant, please remove.

Conclusion – please conclude your work not summarizing it. You can directly point out on that average or range of absorbed dose received by the microorganism based on 3 simulation scenarios in 2-3 lines and conclude that the dose is compatible to threshold limit by ERICA (please use their original recommended levels by IAEA or ICRP), and conclude that the microorganism extremely exposed to DNA damages based on computed mutation rate or DSB or SSB.
